# Molecular Insights into Coumarin Analogues as Antimicrobial Agents: Recent Developments in Drug Discovery

**DOI:** 10.3390/antibiotics11050566

**Published:** 2022-04-24

**Authors:** Rameshwar S. Cheke, Harun M. Patel, Vaishali M. Patil, Iqrar Ahmad Ansari, Jaya P. Ambhore, Sachin D. Shinde, Adel Kadri, Mejdi Snoussi, Mohd Adnan, Prashant S. Kharkar, Visweswara Rao Pasupuleti, Prashant K. Deshmukh

**Affiliations:** 1 Department of Pharmaceutical Chemistry, Dr. Rajendra Gode College of Pharmacy, Malkapur 443101, India; ambhorejp02@gmail.com; 2 Department of Pharmaceutical Chemistry, R. C. Patel Institute of Pharmaceutical Education and Research, Shirpur 425405, India; hpatel_38@yahoo.com (H.M.P.); ansariiqrar50@gmail.com (I.A.A.); 3 Department of Pharmaceutical Chemistry, KIET School of Pharmacy, KIET Groups of Institutions, Delhi-NCR, Delhi 201206, India; 4 Department of Pharmacology, Shri. R. D. Bhakt College of Pharmacy, Jalna 431213, India; sdshinde8390@gmail.com; 5 Faculty of Science of Sfax, Department of Chemistry, University of Sfax, B.P. 1171, Sfax 3000, Tunisia; lulkadel@yahoo.fr; 6 Department of Pharmaceutical Chemistry, Faculty of Science and Arts in Baljurashi, Albaha University, P.O. Box 1988, Albaha 65527, Saudi Arabia; 7 Department of Biology, College of Science, University of Hail, Hail P.O. Box 2440, Saudi Arabia; snmejdi@yahoo.fr (M.S.); drmohdadnan@gmail.com (M.A.); 8 Laboratory of Genetics, Biodiversity and Valorization of Bio-Resources (LR11ES41), University of Monastir, Higher Institute of Biotechnology of Monastir, Avenue Tahar Haddad, BP74, Monastir 5000, Tunisia; 9 Department of Pharmaceutical Sciences and Technology, Institute of Chemical Technology, Matunga, Mumbai 400019, India; ps.kharkar@ictmumbai.edu.in; 10 Department of Biomedical Sciences and Therapeutics, Faculty of Medicine & Health Sciences, University Malaysia Sabah, Kota Kinabalu 44800, Malaysia; 11 Department of Biochemistry, Faculty of Medicine and Health Sciences, Abdurrab University, Pekanbaru 28291, Indonesia; 12 Centre for International Collaboration and Research, Reva University, Rukmini Knowledge Park, Kattigenahalli, Yelahanka, Bangalore, Karnataka 560064, India; 13 Department of Pharmaceutics, Dr. Rajendra Gode College of pharmacy, Malkapur 443101, India; pkdesh@rediffmail.com

**Keywords:** coumarins, antibacterial, SAR, microbial resistance, coumarin hybrids

## Abstract

**Simple Summary:**

Coumarins are a large family of benzopyrones, and more than 1300 coumarins have been reported to date. Natural, as well as synthetic, coumarins have demonstrated a diverse activity spectrum. On the other hand, the demands of the current health scenario witnessing morbidity and mortality due to microbial infections and multidrug-resistant bacterial strains, the well-reported phytochemical coumarin can be of interest. Some of the well-reported coumarin analogues as antimicrobial agents include β-lactum derivatives, coumarin-based 1,2,3-triazole compounds, the miconazole analogue, coumarin-substituted pyrazole hybrids, pyranocoumarin, coumarin−sulphonamide hybrids, pyranocoumarins, coumarin−sulphonamide derivatives, chromenylpyrazoles candidates, 3-amidocoumarins analogues, uracil−coumarin hybrids, indolinedione−coumarin hybrids, coumarin−imidazole hybrids, coumarin-fused pyrazolones and methyl thiazole derivatives, coumarin−theophylline hybrids, etc. In the present review, several methods for the synthesis of coumarin derivatives as antimicrobial agents are reported, along with structure−activity relationship (SAR) studies focusing on the developments reported since 2016.

**Abstract:**

A major global health risk has been witnessed with the development of drug-resistant bacteria and multidrug-resistant pathogens linked to significant mortality. Coumarins are heterocyclic compounds belonging to the benzophenone class enriched in different plants. Coumarins and their derivatives have a wide range of biological activity, including antibacterial, anticoagulant, antioxidant, anti-inflammatory, antiviral, antitumour, and enzyme inhibitory effects. In the past few years, attempts have been reported towards the optimization, synthesis, and evaluation of novel coumarin analogues as antimicrobial agents. Several coumarin-based antibiotic hybrids have been developed, and the majority of them were reported to exhibit potential antibacterial effects. In the present work, studies reported from 2016 to 2020 about antimicrobial coumarin analogues are the focus. The diverse biological spectrum of coumarins can be attributed to their free radical scavenging abilities. In addition to various synthetic strategies developed, some of the structural features include a heterocyclic ring with electron-withdrawing/donating groups conjugated with the coumarin nucleus. The suggested structure−activity relationship (SAR) can provide insight into how coumarin hybrids can be rationally improved against multidrug-resistant bacteria. The present work demonstrates molecular insights for coumarin derivatives having antimicrobial properties from the recent past. The detailed SAR outcomes will benefit towards leading optimization during the discovery and development of novel antimicrobial therapeutics.

## 1. Introduction

Microbial resistance has now become a major health concern and serious challenge for global researchers to address. According to the World Health Organization (WHO), approximately 50,000 men, women and children die of microbial infections around the world every day [1]. A WHO report indicated that some 16 million people died in 1990, which decreased by 1% in 2010 (15 million) and is expected to reach 13 million by 2050. According to the report by the Centers for Disease Control and Prevention (CDCs), more than 2.8 million antibiotic infections occur every year, and more than 35,000 people die in the USA alone because of these infections. One of the CDC’s four recommendations for antibiotic resistance is the development of new antibiotics and diagnostic tests for the treatment of drug-resistant bacteria [2]. Without a question, the number of deaths caused by microbial infections is declining, albeit at a very slow pace, which may be due to the growth in the global population and microbial resistance [3]. The recent focus on the development of antimicrobial agents must address some major issues, like a high risk of toxicity, lack of antimicrobial activity, and development microbial resistance. For a long time, the chemistry of heterocyclic compounds was an interesting field of study to investigate antimicrobial agents [4]. 

**Figure 1:**
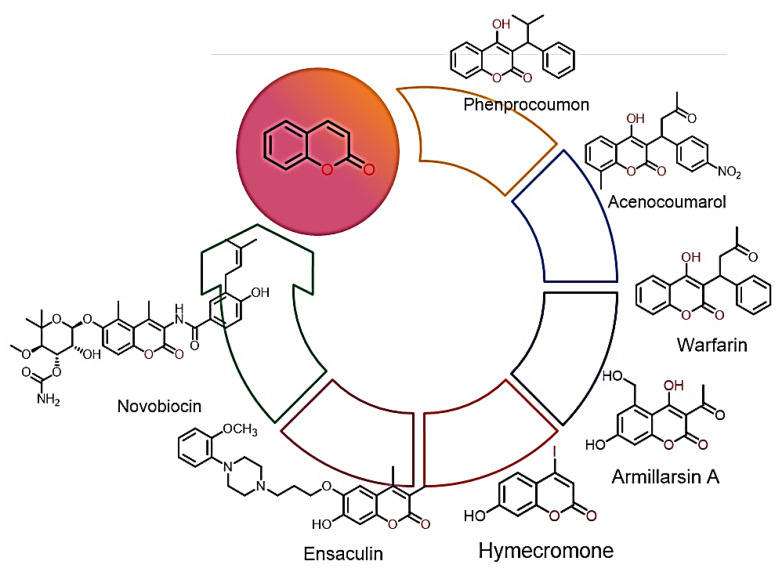
Pharmacological spectrum of coumarins.

Coumarin belongs to the flavonoid class and is found in both natural and synthetic products [5]. Coumarins (1) belongs to heterocyclic α-benzopyrone systems from *Coumarouna odorata* auba (Dipteryx odorata) [6]. It occurs naturally in several plants, microorganisms, and essential oils as a secondary metabolite [7]. Their different pharmacological potentials and less harmful effects on normal cells have made them a hot point in medicinal chemistry. Clinical applications of several these heterocycle members have been widely reported. For example, Warfarin (3), Phenprocoumon (2), and Acenocoumarol (4) are well-known as potent anticoagulant agents [8]; Novobiocin and Armillarisin A are antibiotic [9]; Ensaculin (7) is also a well-known for its antidementia effects [10]; and Hymecromone [11] is a choleretic and antispasmodic agent (Figure 1) [12]. 

This review summarises the current innovations of coumarin-based derivatives with potential antibacterial activity against a range of Gram-negative and Gram-positive bacteria and different aspects of the structure−activity relationship (SAR). The improved SAR gives further insight into the rational improvement of coumarin hybrids, with improved efficacy against multidrug resistance (MDR) bacteria. The guidelines available for antimicrobial susceptibility testing were issued and revised by the Clinical Laboratory Standards Testing (CLSI) and the European Committee on Antimicrobial Susceptibility Testing (EUCAST). Both of them have acceptable levels of agreement, and any of them can be adopted. In the case of a lack of resources, the EUCAST guidelines, being freely available, are preferable [13]. These derivatives can be further categorized as significant/moderate/less active antimicrobials based on their MIC for extracts <100 or 100–625 or >625 μg/mL, respectively for compounds <10 or 10 to 100 or >100 μg/mL, respectively [14]. The edible plant extracts/their MIC ranges are used to distinguish between very active (<100 μg/mL), significantly active (100–512 μg/mL), moderately active (512–2048 μg/mL), and less active (>2048 μg/mL) [15]. Generally, MIC < 100 μg/mL and <10 μg/mL are significantly considerable for extracts and isolated compounds and helpful in categorizing antimicrobial agents [16]. We believe that this review will provide medicinal chemists with a new horizon to look for coumarin-based derivatives that will contribute greatly to drug development.

## 2. Antimicrobial Spectrum of Coumarins

**Scheme 1:**
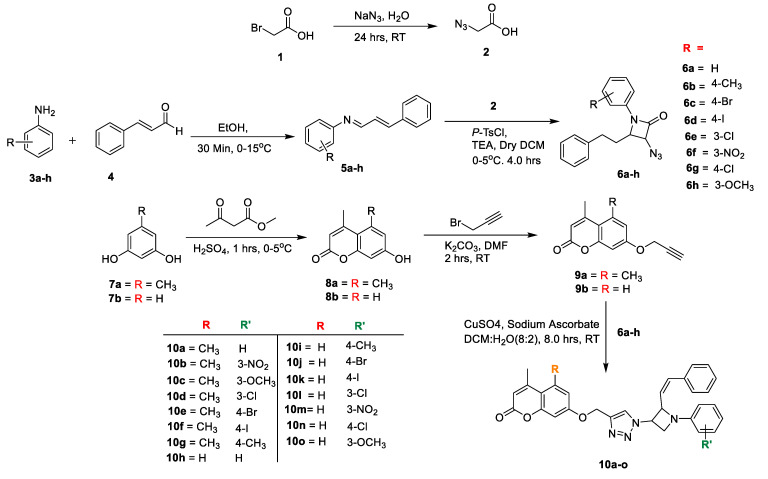
Synthetic route for azido β-lactum derivatives **6**(**a–h**) and coumarin β-lactum derivatives **10**(**a–o**). Reproduced and adapted with permission from Ref. [17]. Copyright 2019, John Wiley and Sons.

Dhawan and co-workers [17] reported the synthesis of hybrid derivatives, i.e., coumarin-clubbed−β-lactam triazole derivatives (**10a–o**), and tested them for their antimicrobial potential against Gram-positive methicillin-resistant *Staphylococcus aureus*, as well as four Gram-negative bacteria, such as *Pseudomonas aeruginosa*, *Klebsiella pneumonia*, *Escherichia coli*, and *Acinetobacter baumannii*, and two fungal strains, like *Candida albicans* and *Cryptococcus neoforman*. An outline for the synthesis of these novel analogues is depicted in Scheme 1. The construction of the target hybrids (**10a–o**) began by the reaction of 7-(ethynyloxy)-4,5-dimethyl-2H-chromen-2-one (**9a**) and 7-(ethynyloxy)-4-methyl-2H-chromen-2-one (**9b**) with various synthesized substituted azido lactams (**6a–h**) in the presence of copper sulphate pentahydrate and sodium ascorbate in a ratio of dichloromethane:water (8:2) at room temperature. The starting materials **8a** and **8b** were synthesized by a reaction of orcinol **7a** and phloroglucinol **7b** with ethyl acetoacetate in sulphuric acid via the Pechmann rection. Compound **9a** was obtained by the base-promoted reaction of **8a** with propargyl bromide in dimethylformamide (DMF). Substitute azido lactams were synthesized by a reaction with acetic acid and imine at 0–5 °C. Substituted key intermediate azidoketene (**6a–h**) were prepare in situ in the presence of p-toluene sulfonyl chloride and triethylamine to furnish the desired azido lactams. The antibacterial potency of the furnished compounds was detected by the disc diffusion method at a conc. Of 32 µg/mL with ≤ 1% DMSO. From the displayed results, most of compounds were moderately active against all bacterial excerpts and fungal stains. Further studying revealed compounds **10c**, **10f**, and **10g** possess significant activity against *Staphylococcus aureus*, with maximum percentage inhibitions of 7.75, 7.92, and 8.94%, respectively, while the other analogues were inactive with methicillin-resistant *Staphylococcus aureus* (MRSA), whereas compounds **10i**, **10j**, **10k**, and **10d** emerged as active hybrids against *C. albicans* with 21.65, 9.42, 6.24, and 14.96 percentage inhibition, respectively. The SAR study revealed analogues bearing methyl and iodo groups showing significant antimicrobial activity.

**Scheme 2:**
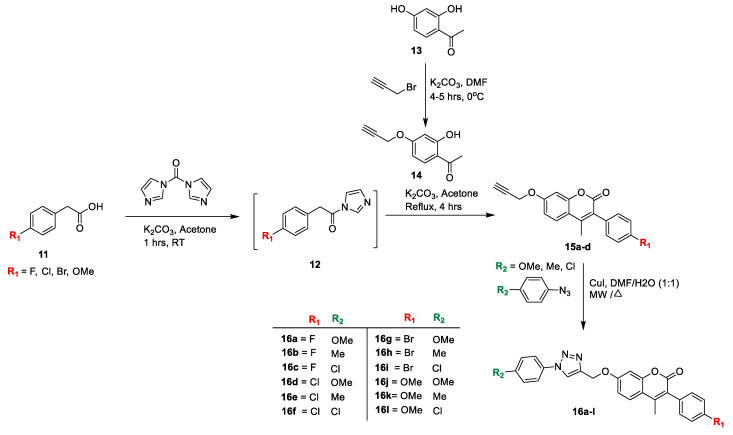
Synthetic route for coumarin—based 1,2,3—triazole compounds **16**(**a–l**). Reproduced and adapted with permission from Ref. [18]. Under Creative Commons CC BY NC 3.0. Copyright 2020, Royal Society of Chemistry.

Dharavath and co-workers [18] reported a set of coumarin-based 1,2,3-triazole hybrids **16**(**a–l**) and tested for their antibacterial activity towards Gram-positive, such as *S. aureus* and *Bacillus subtilis*, and Gram-negative bacteria like *E. coli*, as well as *K. pneumonia*. The route for the synthesis is outlined in Scheme 2 [14]. The substituted phenyl acetic acid **11** was activated by 1,1-carbonyldiimidazole and potassium carbonate as a base in acetone for 1 h to furnish intermediate **12**. Later, compound **14** was prepared by the selective propargylation of **13** using K_2_CO_3_ in DMF as a solvent. Prepared alkyne-substituted hydroxy acetophenone **14** was refluxed with intermediate **12** using K_2_CO_3_ and acetone to obtain the key intermediate (**15a–d**). In a last step, the title analogues (**16a–l**) were afforded a copper (I)-catalyzed Huisgen cycloaddition reaction of intermediate **15a–d** with different aryl azides using copper iodide and DMF/H_2_O (1:1). The antibacterial and antifungal potentials of the furnished analogues detected by the disc diffusion method and percentage zone inhibition were compared to standard drugs Gatifloxacin and Clotrimazole, respectively. Among compounds **16a**, **16d**, **16g**, and **16j** possess potent antibacterial action due to methoxy group in the triazole moiety was shown, while compounds,**16b**, **16c**, **16e**, **16f**, **16h**, and **16i** showed significant potential against the tested organisms because of electron-withdrawing groups on the coumarin ring when compared to the standard drug.

**Figure 2:**
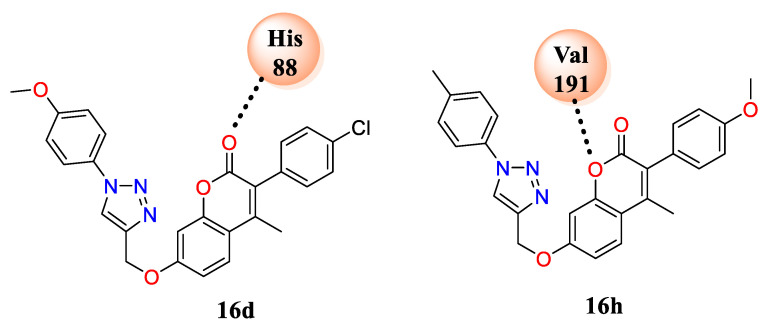
Docking images of compounds **16d** and **16h** showing H-bond interactions with 2VCX protein.

Antifungal potential of the tested compound measured against *Aspergillus niger*, *Aspergillus flavus*, and *Fusarium sporum* at a concentration of 50 mg mL^−1^. Among the screened hybrids **16a–c** displayed significant potential against all tested fungal strains due to the existence of fluorine on the coumarin ring, while compounds **16j–l** showed good potential due to the appearance of -OCH_3_ in the triazole ring. The docking study clearly showed the interaction profile between the coumarin-based 1,4-disubstituted 1,2,3-triazole moiety with amino acids. Active analogues **16d** and **16h** formed H-bond interactions with His-88, Val-191, and amino acids with water in the cavity of the selected proteins, i.e., DNA-gyrase B and topoisomerase IV (PDB ID: 4GEE) and cytochrome P450 EryK (PDB ID: 2XFH) (Figure 2). When the results were compared to the well-reported antifungal agent ketoconazole (0.5 μg/mL against *A. niger*), the above coumarin-based 1,2,3-triazole hybrids were not potentially active [18].

**Scheme 3:**
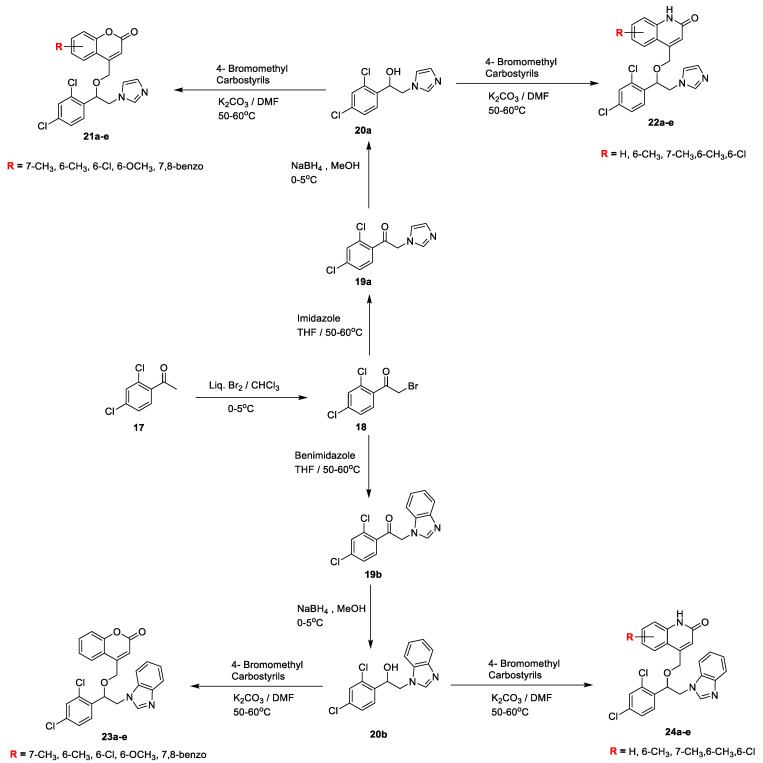
Synthetic route for coumarin and 1–aza–coumarin–linked miconazole analogues. Reproduced and adapted with permission from Ref. [19]. Copyright 2020, John Wiley and Sons.

S.M. Sutar et al. [19] performed the synthesis of coumarin and 1-aza-coumarin derivatives of miconazole (**21a–e**), (**22a–e**), (**23a–e**), and (**24a–e**) and evaluate there *in vitro* antimicrobial potential. The route for their synthesis is outlined in Scheme 3. Initially,2-bromo-1-(2,4-dichlorophenyl) ethenone **18** was obtained via the bromination of **17**. Further, corresponding with **18** in the reaction with imidazole and benzimidazole in THF under reflux furnished N-alkyl analogues **19a** and **19b**. Key intermediates **20a** and **20b** were afforded via a reduction of **19a** and **19b** using NaBH_4_. Finally, the O-alkylation of **20a** and **20b** with various substituted 4-bromomethyl coumarins and 4-bromomethyl carbostyrils using K_2_CO_3_ and DMF yielded target compounds **21a–e**, **22a–e**, **23a–e**, and **24a–e**, respectively.

**Figure 3:**
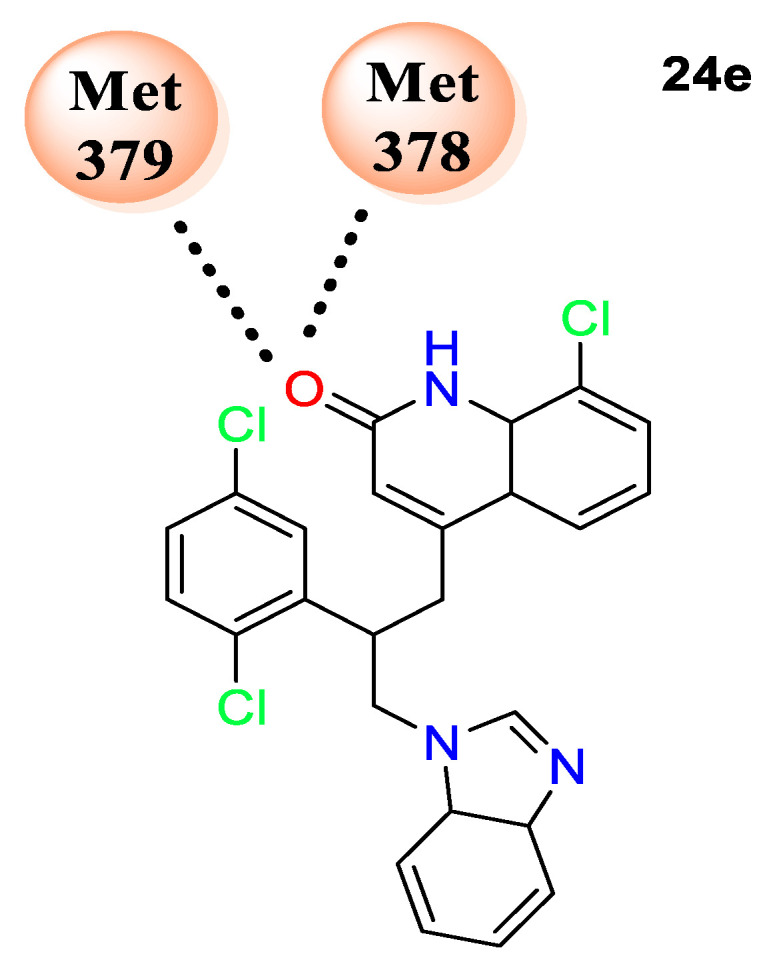
Two-dimensional representation of the interactions of **24e** in the active site 3LD6 complex.

Among the series, majority of the analogues were found to be moderate-to-significantly active against Gram-positive strains, such as *S. aureus* and *Bacillus cereus*, whereas derivatives of miconazole (**21a–c**) displayed reflective inhibitory activity against both Gram-positive strains, with MIC ranging from 1 to 4 μg/mL. Likewise, the replacement of imidazole with benzimidazole analogues (**23a–c**) revealed the same potency as compared to **21a–c**. The MIC value of the tested analogues explored that presence of electron-donating species like methyl **21a**, **21b**, **23a**, and **23b** and methoxy **21c** and **23c** on coumarin employed excellent potency as compared to an electron-withdrawing group (ex. Chloro and aryl ring). Therefore, analogues **21a** and **23c** against *B. Cereus* and analogue **23c** against *S. aureus* displayed 100% potency (1 μg/mL) compared to the control ciprofloxacin (1 μg/mL); however, compounds **21a**, **21c**, and **23b** against *S. aureus* and compounds **21b**, **21c**, **23a**, and **23b** against *B. Cereus* recorded 50% potency (2 μg/mL) when compared to ciprofloxacin. Two groups of fungal strains, i.e., Yeast (included *C. albicans*, *Candida utilis*, and *Candida krusei*) and Filamentous (including *Aspergillus fumigatus*, *A. niger*, and *Rhizoctonia bataticola*) fungi, were screened against the synthesized analogues and their potential compared with the standard drugs Itraconazole and Miconazole. None of analogues proved better than that of the standard drug against *A. niger* and *R. bataticola*, excluding **23e** against *A. niger* and **23c** against *R. bataticola*. However, the compounds bearing benzimidazole (**23a–24e**) exhibited good-to-moderate potential (4–31.2 μg/mL) against *A. fumigatus* compared to that of the compounds bearing an imidazole framework (**21a–22e**). Candidates having an aza-coumarin framework with methyl and chloro substituents (**24b–e**) showed better potency (4 μg/mL). In conclusion, the majority of candidates were found effective against the inhibition of all fungi species; in particular, **21e–24e** were remarkable, while analogue **21e** of the 7,8-benzo substitution on coumarin recorded 4 μg/mL of MIC, and the replacement of coumarin with 1-aza-coumarin (**22a–e**) displayed 200−400% efficiency against all three strains. The molecular docking study revealed that compound **24e** displayed the highest binding affinity of 11.2 kcal/mol with protein human lanosterol 14α-demethylase (PDB ID: 3LD6) by forming hydrogen-bonding interactions with amino acids MET 378 and ILE 379 (Figure 3). 

**Scheme 4:**
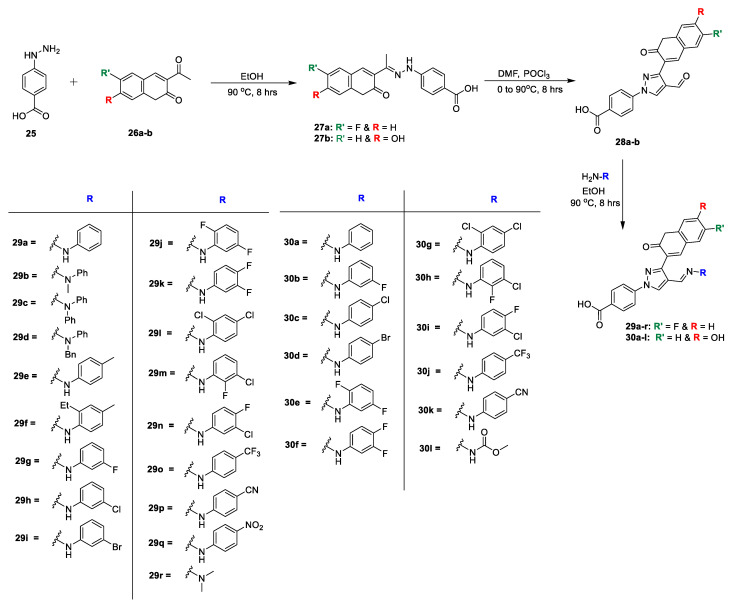
Synthesis of coumarin—substituted pyrazole—derived aldehyde (**29a–r**) and hydrazones (**30a–l**). Reproduced and adapted with permission from Ref. [20]. Under Creative Commons CC BY 4.0. Copyright 2020, MDPI.

Rawan Alnufaie and co-workers [20] reported the synthesis of some coumarin-substituted pyrazole hybrids for antimicrobial evaluation against methicillin-resistant *S. aureus*. 4-hydrazino benzoic acid **25** refluxed with fluoro **26a**, and hydroxy **26b** bearing 3-acetylcoumarin furnishes corresponding to hydrazones **27a** and **27b**, respectively, which was subjected to reflux using POCl_3_/DMF to yield formyl-substituted pyrazole derivatives **28a–b**. Intermediate fluoro-substituted coumarin **28a** and hydroxy-substituted coumarin derivative **28b** reacted with substituted phenyl hydrazine under reflux to obtain coumarin-substituted pyrazole-derived hydrazones **29a–r** and **30a–l** outlined in Scheme 4. Antibacterial potency of the synthesized analogues was detected against eight types of Gram-positive and three Gram-negative bacterial strains. Compound **29c** with *N*,*N*-diphenyl substitution has a proven potential against all Gram-positive bacterial strains. Compound **29c** inhibited the growth of methicillin-sensitive *S. aureus* with the lowest MIC of 3.125 μg/mL, as well as four methicillin-resistant strains also inhibited by this candidate (MIC = 1.56 μg/mL). A compound bearing chloro (**29h**) and bromo (**29i**) substitutions displayed moderate activity against Gram-positive strains. This candidate was found to be active against all three *A. baumannii* strains (MIC = 6.25 μg/mL). The candidate having 3,4-difluro substitution (**29k**) was found moderately potent against both Gram-positive and Gram-negative bacterial strains with a MIC of 6.25 μg/mL. Candidates with very strong electron-withdrawing groups like trifluoromethyl (**29o**) were found significantly active against Gram-positive strains with a MIC value of 3.125 μg/mL, while the other two analogues **29p** and **29q** with very strong electron-withdrawing frameworks were inactive.

Hydroxy-substituted coumarin analogues **30a–l** failed to show antibacterial potential against all tested strains. Based on the observed results, the SAR study revealed that compounds bearing a fluoro substitution on coumarin produced potent antimicrobial action, while the hydroxy-substituted coumarin analogues almost eliminated the antimicrobial potential. This may happen due to the different natures of hydroxy and fluoro substituents. Candidates with fluoro and *N*,*N*-diphenyl substitutions possessed significant antimicrobial action, but one of the phenyls from the diphenyl substitution when replaced with methyl (**29b**) or benzyl (**29d**) species eliminated the potential of the compounds. Hydrophobic mono-substituted analogues were found to possess better potential than that of the disubstituted analogues. A compound with a trifluoromethyl framework (**29o**) was found the most potent among all the synthesized compounds against the Gram-positive bacterial strains. 

**Scheme 5:**
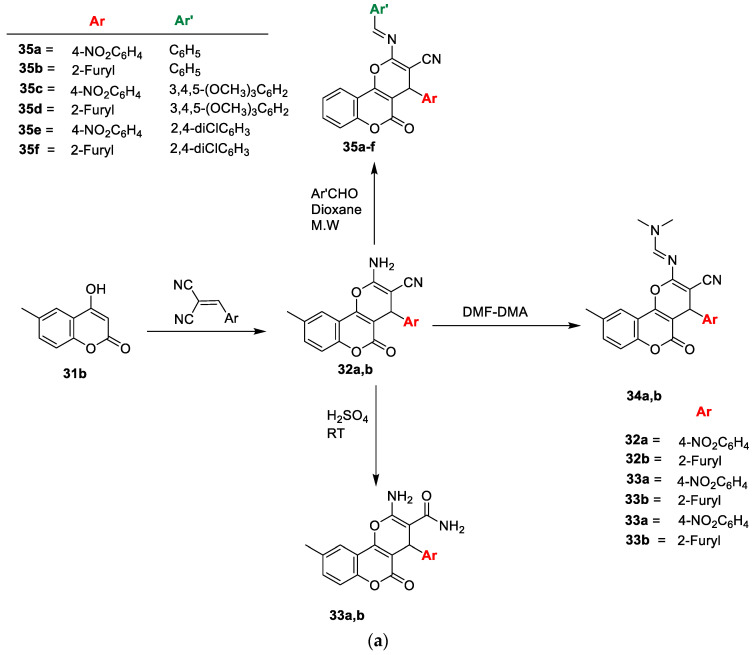

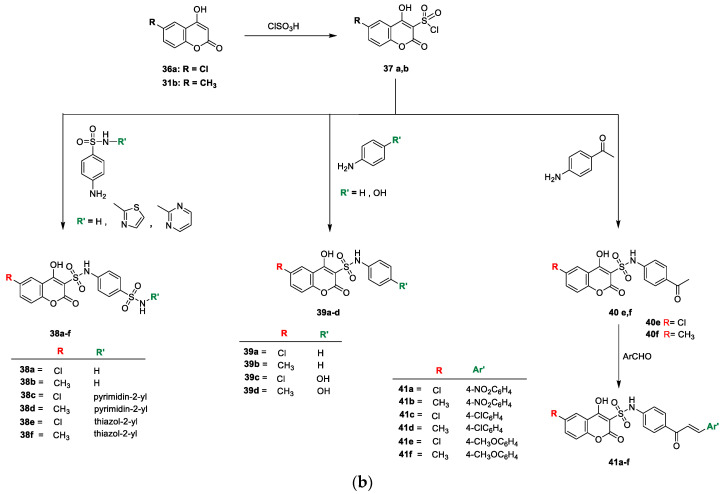
(**a**) Synthetic routes of pyranocoumarins. (**b**) Synthetic routes of coumarin—sulphonamide derivatives. Reproduced and adapted with permission from Ref. [21]. under Creative Commons CC BY 4.0. Copyright 2020, MDPI.

Hanan M. Alshibl and colleagues [21] stated the antimicrobial potential of the pyranocoumarin and coumarin−sulphonamide hybrids (Scheme 5a,b). Initially, new pyranocoumarins (**32a,b**) were furnished via one-pot synthesis by the reaction of substituted aryl aldehyde with malononitrile and **31b** in the presence of potassium hydrogen phthalate. The carboxamide analogues **33a** and/or **33b** were prepared by the acid hydrolysis of **32a** and/or **32b**, respectively, with sulphuric acid. Similarly, the reactions of **32a** and **32b** with excess N, N-dimethylformamide-dimethylacetal afforded the corresponding **34a** and **34b**. Target analogues **35a–f** were yielded by the reaction of intermediate **32a,b** with various substituted aromatic aldehydes in 1,4-dioxane. Coumarin sulfonyl chloride **37a,b** was prepared by reacting 4-hydroxy-6-substituted coumarin **36a** and/or **31b** with chlorosulfonic acid in DCM. Coumarin−3-sulphonamides (**38a–f**) were obtained by refluxing **37a** or **37b** with substituted sulpha analogues. Similarly, **37a** or **37b** in a reaction with the nucleophilic agent aniline and p-substituted aniline in EtOH under reflux furnished the corresponding coumarin−3-sulphonamides (**39a–d** and **40e–f**, respectively). Coumarin−sulphonamide chalcone analogues **41a–f** were prepared via the Claisen–Schmidt condensation of *p*-acetyl derivatives (**40e** or **40f**) with different *p*-substituted aromatic aldehydes by the addition of NaOH in absolute ethanol (Scheme 5b). Synthesized analogues were screened for their in vitro antimicrobial potential against Gram-positive bacterial strains, namely, *S. aureus*, *B. subtilis*, *Bacillus*, and *Megaterium*, and Gram-negative strains such as *E. coli* and *P. aeruginosa*. Candidates were also screened against yeast and yeast-like pathogenic fungal strains *Saccharomyces cerevisiae* and *C. albicans*, respectively. Screening was accomplished by using agar well diffusion and Sabouraud dextrose agar. Among the synthesized analogues, **32a**,**b**, **35a**,**c,f**, **38a**,**c**,**d–f**, **39a**,**c**,**d**, and **41b–f** were found to be potent antimicrobial agents with Izs larger than 25 mm against at least one tested microbial strain, while compounds **35e** and **39b** displayed moderate potential with Izs between 20 and 24 mm. However, **33a**,**b, 34a**,**b**, **35d**, **39e–f**, and **41a** exhibited marginal activity with Izs 15–19 mm, whereas analogues **38c**,**d 39c,d**, and **41c,d** displayed the maximum Izs and revealed potent antimicrobial action towards the tested microorganisms when compared to the reference with a MIC of 125 µg/mL. However, **38d** established remarkable broad-spectrum potential towards all the tested microorganisms with a MIC of 125 µg/mL. Analogues bearing sulfadiazine or 4-hydroxyphenyl moieties, **38c–d** and **39c–d**, revealed fairly excessive antibacterial potential (30 mm) compared to the standard ciprofloxacin (28 mm) against *S. aureus* with a MIC of 125 µg/mL, while analogues containing sulphanilamide or 4-chlorophenyl substitutions (**38a** and **41c,d**) exhibited equal antibacterial potential against *S. aureus* than the standard analogue ciprofloxacin (28 mm).

**Scheme 6:**
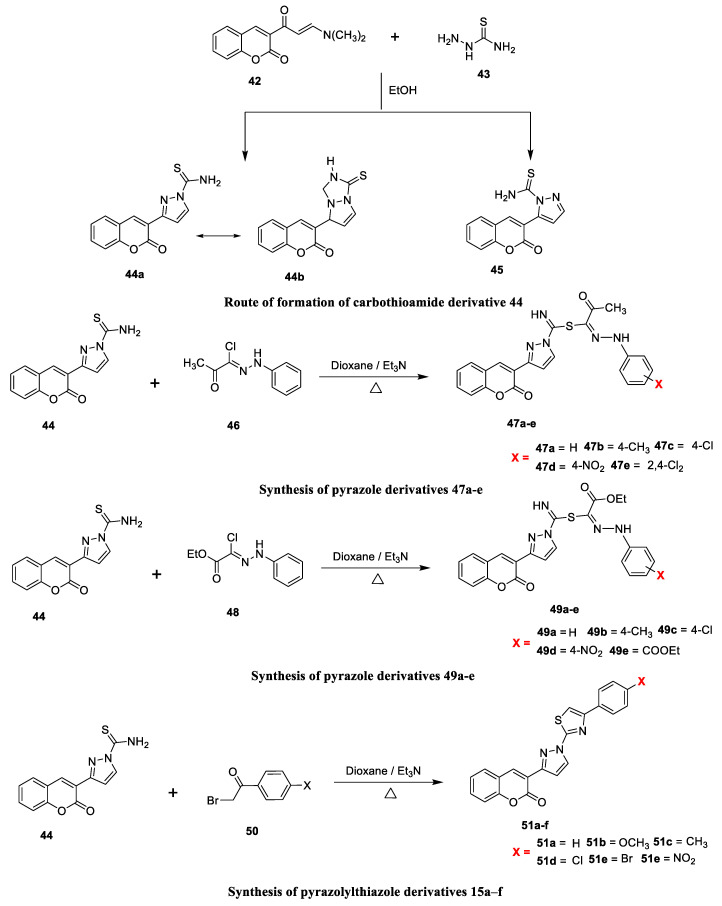
Outline for the synthesis of the chromenyl—pyrazoles candidates. Reproduced and adapted with permission from Ref. [22]. Copyright 2020, John Wiley and Sons.

Abrar Bayazeed and co-workers [22] reported the antimicrobial potency of novel coumarin derivatives obtained from 3-(2-oxo-2H-chromen-3-yl)-pyrazole-1-carbothioic acid amide with two diverse hydrazonoyl chlorides and phenacyl bromides when screened against fungal and bacterial strains. The synthesis of the target analogues is outlined in Scheme 6. The key intermediates of carbothioamide (**44** and **45**) were furnished via condensation and Michel-type addition succeeded by eliminating water and Me_2_NH by a reaction of enaminone (**43**) and thiosemicarbazide (**42**). Key intermediate carbothioamide (**44**) afforded chromen-3-yl-pyrazole derivatives **47a–e** and **49a–e** via a substitution reaction with two different types of hydrazonoyl chlorides, **46a–e** and **48a–e**, respectively. Finally, thiazole derivatives **51a–f** were prepared via the nucleophilic substitution reaction of the intermediate carbothioamide (**44**) with substituted phenacyl bromide under reflux. The synthesized chromenylpyrazoles candidates were examined in vitro for their antimicrobial potential versus two fungal Gram-positive and Gram-negative bacterial strains by using the agar diffusion method.

All test compounds were examined separately against all microbial strains at a concentration of 30 μg/mL. Among the series, four analogues, namely, **49e**, **51b**, **49b**, and **51c**, showed an exceeded potential compared to that of the reference/standard ketoconazole against *A. fumigatus* with percent zone inhibitions of 164%, 158.8%, and 147.1%. Moreover, two analogues, **49b** and **51c**, showed excellent activity against *C. albicans* above the reference ketoconazole 1.5-fold, while compounds **47b**, **49e**, and **51b** exhibited good potency as compared to ketoconazole against *C. albicans* with inhibition zone percentages 75–85%. The SAR studies stated that the presence of two ester groups in the synthesized derivatives is responsible for the improved antifungal potential, as seen in **49e**. Moreover, analogues bearing electron-donating groups CH_3_ and OCH_3_ at the *para* position of the aromatic skeleton (**51b**, **49b**, and **51c**) had improved potential, and it exceed that of the reference drug. The antibacterial potential compounds **49b** and **51c** were found highly potent against *S. aureus* with inhibition percentages 104.2% and 104%, respectively. Two other analogues included in the study, namely, **49e** and **51b**, exhibited the same excellent potential as compared to the reference Gentamicin with 107.7% and 103.8% inhibition. The Gram-negative bacteria *K. pneumoniae* was highly affected by analogues **47a** and **49e** with 71.4% inhibition, while most of the analogues were found less active against *E. coli*. The SAR analysis also revealed that, for the inhibition of Gram-positive bacteria, the presence of ester and electron-donating substitution at the *para* position of the aromatic framework showed antimicrobial potency, as observed for analogues **49e**, **49b**, **51c**, and **51b** [22]. 

**Scheme 7:**
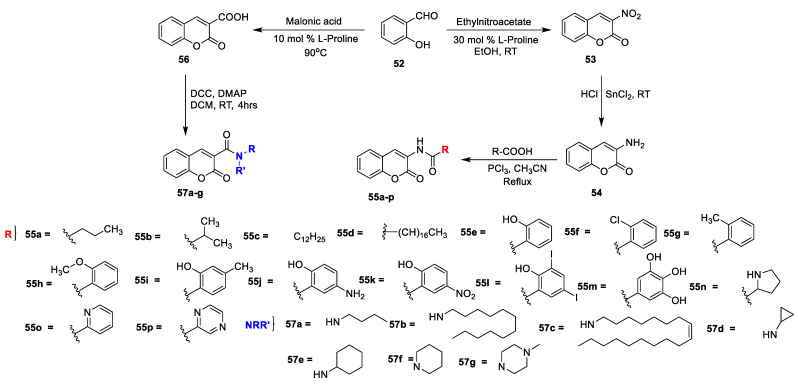
Route for the synthesis of 3—amidocoumarins. Reproduced and adapted with permission from Ref. [23]. Copyright 2020, Elsevier.

**Figure 4:**
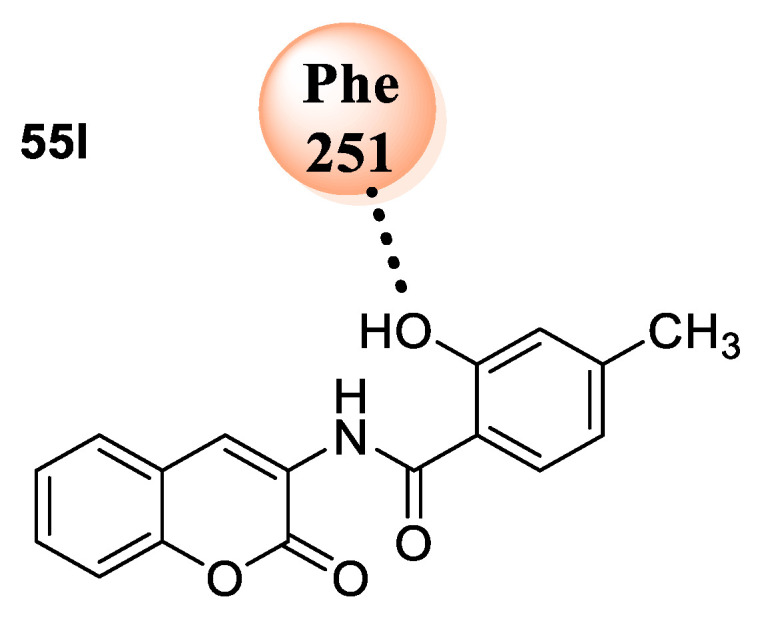
Compound **55l** docked within the active site of 1W9U.

R K Sharma et al. [23] synthesized a set of 3-amidocoumarins analogues and evaluated their in vitro antimicrobial action against four Gram-negative, two Gram-positive bacterial, and three fungal pathogens. The synthesis of the designed analogues is depicted in Scheme 7. In this study, 3-nitrocoumarin (**53**) was obtained via the condensation of salicylaldehyde (**52**) and ethyl nitroacetate in the presence of L-proline as a catalyst. Later, the reduction of **53** to 3-aminocoumarin (**54**) using SnCl_2_ subsequently by acylation with different substituted carboxylic acids using PCl_3_ in acetonitrile under reflux furnished the target analogues of 3-amido coumarins **55a–p**. On the other hand, coumarin-3-carboxylic acid **56** synthesized via the Knoevenagel condensation of salicylaldehyde (**52**) with malonic acid in the presence of L-proline followed by the condensation of synthesized coumarin−3-carboxylic acid (**56**) with suitable amines using DCC and DMAP in dry DCM gave the corresponding 3-carboxamide coumarins **57a–g**. The MIC of amido−coumarins **55a–p** and **57a–g** detected against Gram-negative *Salmonella typhi*, *P. aeruginosa*, *K. pneumoniae*, and *E. coli*; two Gram-positive (*S. aureus* and *Bacillus pumilus*); and three fungal strains (*Candida tropicalis*, *C. albicans*, and *Aspergillus fumigatus*) were determined by the two-fold serial dilution method using novobiocin and chloramphenicol as the antibacterial and fluconazole and amphotericin B as the antifungal standards. Among the series compounds, **55e–f** with hydroxyl, chloro, methyl, and methoxy group substitutions on the phenyl ring demonstrated high MIC in the range of 50 to >200 µg/mL. Some enhancement in the potency was noticed upon the addition of disubstituted phenyl rings in analogues like **55i**, **55j**, and **55k**. Analogue **55i** inhibited the growth of *S. typhi* and *P. aeruginosa* at concentrations of 12.5 and 25 µg/mL, respectively, while analogue **55j** bearing amino and hydroxy groups as substitutions on the phenyl ring exhibited their inhibitory potential against *S. typhi* and *S. aureus* with a MIC value of 25 µg/mL. Enhancement in the potency was shown upon the insertion of an electron-withdrawing nitro group in the place of amino group **55k** against three bacterial strains with MIC 12.5–25 µg/mL. However, analogue **55l** with a diiodo and hydroxyl group at the phenyl ring explored a significant antibacterial potential against *S. typhi*, *S. aureus*, *E. coli*, and *B. pumilus* at lower MIC (6.25–25 µg/mL). Compounds bearing a long alkyl chain dodecyl group (**57b**) and oleyl group showed good inhibitory potential against the *S. typhi*, *E. coli*, and *S. aureus* bacterial strains, whereas compound **57f** with a piperidinyl ring displayed excellent inhibitory potential against *P. aeruginosa*, *S. typhi*, *E. coli*, and *S. aureus* bacterial strains with MIC 6.25–25 µg/mL. If we look at the antifungal potential, analogues **55l**, **57b**, **57c**, and **57f** displayed significant potency against *C. albicans* and *A. fumigatus*, with MICs in the range of 6.25–25 μg/mL as compared to standard drugs, while other analogues from the series exhibited poor activity, with MIC in the range of 50 to >200 μg/mL against the fungal strains. The structure−activity relationship (SAR) has stated that compounds bearing a hydroxy group along with electron-donating methyl group **55i**, amino group **55j**, and, similarly, electron-withdrawing nitro **55k** and diiodo **55l** on the phenyl ring exhibited higher antibacterial potential, with MIC ranging from 6.25 to 25 μg/mL as compared to the analogues with a monosubstituted phenyl group (**55i–l** vs. **55e–h**). This may happen due to di- and tri-substituted phenyl frameworks, which possibly make additional intermolecular interactions leading to the improved penetration of the compounds through the bacterial membrane. Among the series compounds, **55l**, **57b**, and **57f** were identified as the most promising analogues and exhibited broad-spectrum antimicrobial activity. For better understanding, molecular docking studies of the synthesized analogues was performed against *A. fumigatus* chitinase (PDB ID: 1W9U). Compound **55l** explored the highest binding affinity toward a protein with a binding energy −8.44 Kcal/mol. It showed van der Waals interactions with hydrophobic, as well as polar, residues in the binding pocket of the selected protein (Figure 4).

**Figure 5:**
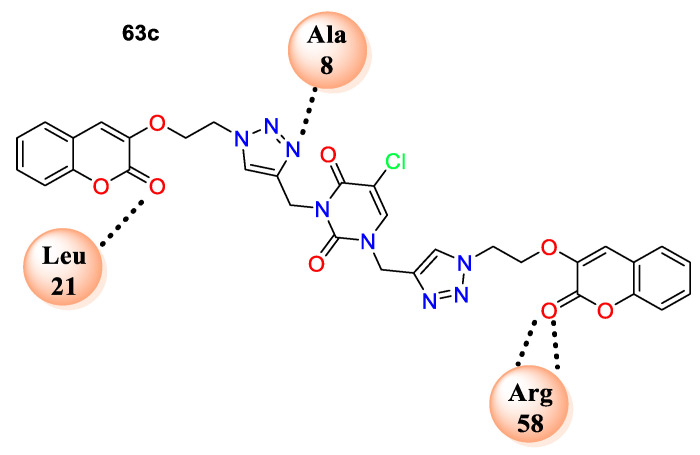
Two-dimensional representation of compound **63c** with a binding site of *S. Aureus* dihydrofolate reductase (PDB ID: 3SRQ).

**Scheme 8:**
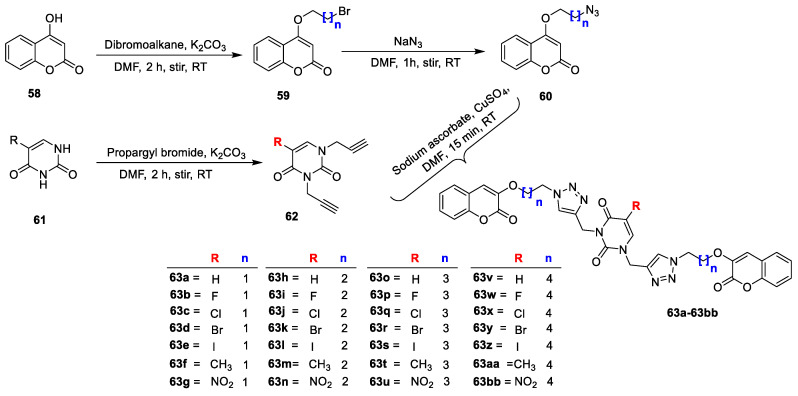
Synthesis of uracil—coumarin hybrids. Reproduced and adapted with permission from Ref. [24]. under Creative Commons CC BY-NC-ND 4.0. Copyright 2019, Elsevier.

Mohit Sanduja et al. [24] designed and synthesized a series of uracil−coumarin-based bifunctional molecular hybrids roped by a 1,2,3-triazole moiety. All the synthesized derivatives were screened for their in vitro antibacterial potency against the *E. faecalis* and *S. aureus* (Gram-positive) and *P. aeruginosa* and *E. coli* (Gram-negative) bacterial strains using the agar well diffusion and broth microdilution methods. Target hybrids were synthesized by starting with 4-hydroxy coumarin (**58**) on a treatment with dibromoalkanes in the presence of K_2_CO_3_ in DMF to yield the desired alkylated coumarins (**59**). Obtained intermediate **59** on further treatment with NaN_3_ in DMF gave azide-substituted coumarins (**60**). Additionally, 5-substituted uracils (**61**) on a treatment with propargyl bromide in the presence of K_2_CO_3_ furnished propargylated uracil analogue **62**. Finally, the alkylated coumarins (**60**) on a reaction with the propargylated uracil analogue (**62**) in the presence of copper sulphate and sodium ascorbate afforded target triazole-linked uracil-coumarin hybrids (**63a,b**), and it is outlined in Scheme 8. Among this series of analogues, **63b** and **63c** were found to possess promising activity against *S. aureus* with a zone of inhibition 26 mm and 28 mm. Moreover, both hybrids exhibited equipotent activity against *Enterococcus faecalis* and *P. aeruginosa* and were found less active against *E. coli*. The MIC value of both the potent hybrids was determined by the broth microdilution method against *S. aureus* and found to be 7.23 lg/mL for **63b** and 11.7 lg/mL for **63c**, which was compared to the standard drug levofloxacin (3.12 lg/mL). The SAR was defined for the compounds with electronegative species on the uracil skeleton, and it favoured an antimicrobial potency. The analogues with -F (**63b**) and -Cl (**63c**) found similar and activity. Moreover, the chain length in between the triazole and coumarin moieties also affected the potency. The analogues with two carbon chain lengths were found potent compared to the candidates with chain lengths with three, four, and five carbons. The docking study demonstrated that compound **63c** fit well in the cavity of dihydrofolate reductase (DHFR) (PDB ID: 3SRQ). One of the coumarin rings of **63c** formed hydrophobic interactions with residues Leu55, Leu29, and Val32, while a second coumarin moiety formed interactions with residues Gly95, Ile15, and Leu21. The triazole ring nitrogen exhibited hydrogen-bonding interactions with the backbone nitrogen of Ala8, as well as strong *p*−*p* stacking interactions observed in between the triazole moiety and Phe93, while a second triazole skeleton showed *p*−*p* stacking interactions with Phe99 (Figure 5). 

**Scheme 9:**
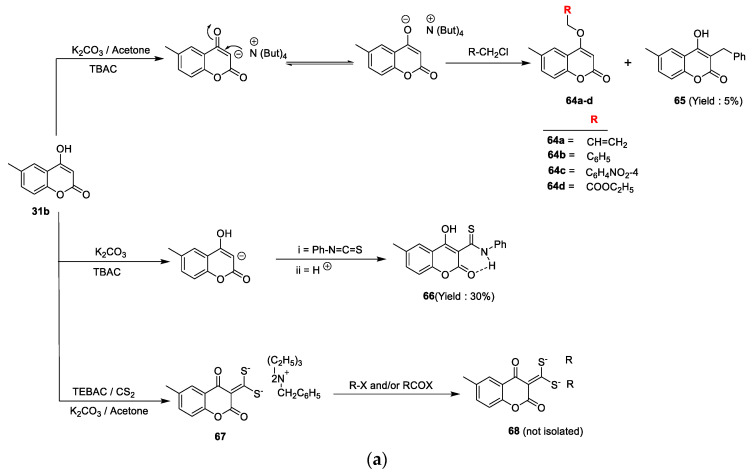

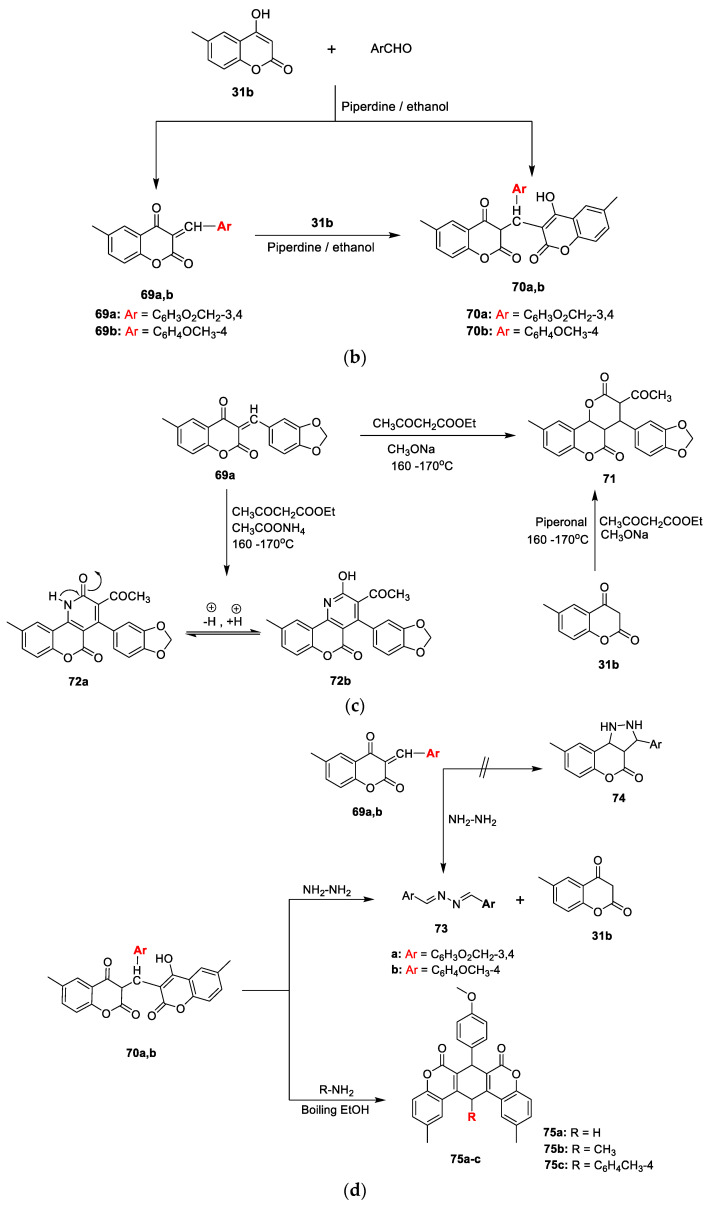
(**a**) PTC reaction of coumarin derivative **31b** with alkyl halides, phenyl isothiocyanate, and CS_2_. (**b**) Reaction of coumarin derivative **31b** with aromatic aldehydes. (**c**) Michael cycloaddition reactions of coumarin derivative **31b** with ethyl acetoacetate. (**d**) Reaction of 3-arylidenes **69a**,**b** and/or dicoumarols **70a**,**b** with nitrogen nucleophiles. Reproduced and adapted with permission from Ref. [25]. Copyright 2020, John Wiley and Sons.

Mohsen K.A. Regal et al. [25] synthesized some new coumarins and dicoumarol derivatives to screen for their in vitro antibacterial potency against Gram-negative *E. coli*, Gram-positive *S. aureus*, and antifungal activity detected against *C. albicans* using the disc diffusion method at a 1 mg/mL concentration. Ampicillin was used as the standard drug for antibacterial and clotrimazole for antifungal action. The reported derivatives were obtained by reacting coumarin **31b** with different alkyl halides under phase transfer catalysis conditions using K_2_CO_3_ as the base and TBAC as the catalyst. This oxygen alkylation through nucleophilic displacement furnished analogues **64a–d** and a smaller amount of compound **65** in the case of benzyl chloride. On the other hand, coumarin **31b** on the treatment with phenylisothiocyanate via the C_3_ addition on the carbon nitrogen double bond of the isothiocyanate yielded 3-(N-phenyl) thiocarbamide coumarin derivative **66**. Similarly, coumarin **31b** via a one-pot three-component phase transfer catalysis reaction converted into intermediate **67** by a reaction with carbon disulphide and different alkyl halides and/or aroyl halides under K_2_CO_3_ as the base and TBAC as the catalyst (Scheme 9a). Coumarin **31b** on reaction with aromatic aldehydes such as 3,4-methylene dioxybenzaldehyde and/or 4-methoxy benzaldehyde in an equimolar ratio via condensation gave 3-arylidene-6-methyl-4-oxocomarin **69a**,**b** under base catalyst piperidine. Repeated reactions using an excess of **31b** could yielded the dicoumarol analogues **70a,b** in good yields (Scheme 9b). Compound **69a** underwent Michael-type cycloaddition reactions and afforded cycloaddition products of pyranochromene (**71**) and pyrano pyridone (**72**) when reacted with ethyl acetoacetate in the presence of methoxide and/or ammonium acetate as the base under fusion conditions (Scheme 9c). The reaction of 3-arylidenes (**69a**,**b**) and/or dicoumarols (**70a**,**b**) with hydrazine hydrate in boiling ethanol furnished the corresponding 1,2-dibenzyldene hydrazine derivatives (**73a**,**b**), whereas **70b** converted to pyridine derivatives **75a–c** via a cyclocondensation reaction of two carbonyl groups with nitrogen nucleophiles by treatment with ammonium acetate, methyl amine, and/or *p*-toluidine under reflux (Scheme 9d). From the set of synthesized analogues, 81% were found to be active against all tested microorganisms. Compounds **64a**, **64b**, **66**, **70a**, **70b**, **71**, and **72** were proven to be promising results against antibacterial and antifungal potential with activity-indexed values ranging between 50% and 87%. The minimum potential was shown by analogues **64c,d** with an activity-indexed value of 40%. 

**Scheme 10:**
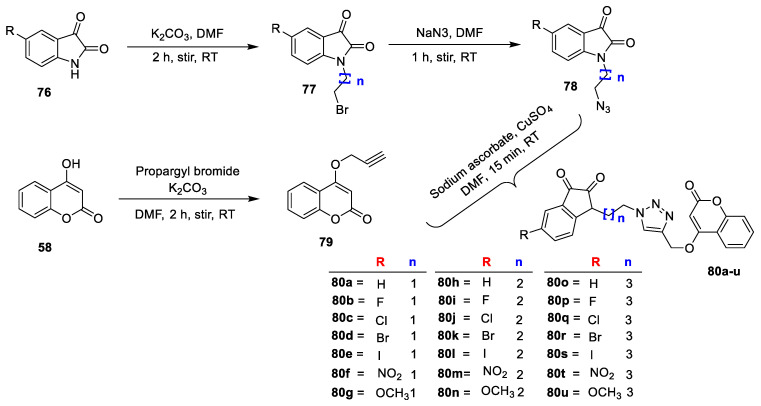
Synthesis of the novel indolinedione—coumarin hybrids **80a–u**. Reproduced and adapted with permission from Ref. [26]. under Creative Commons CC BY-NC-ND 4.0. Copyright 2019, American Chemical Society.

**Figure 6:**
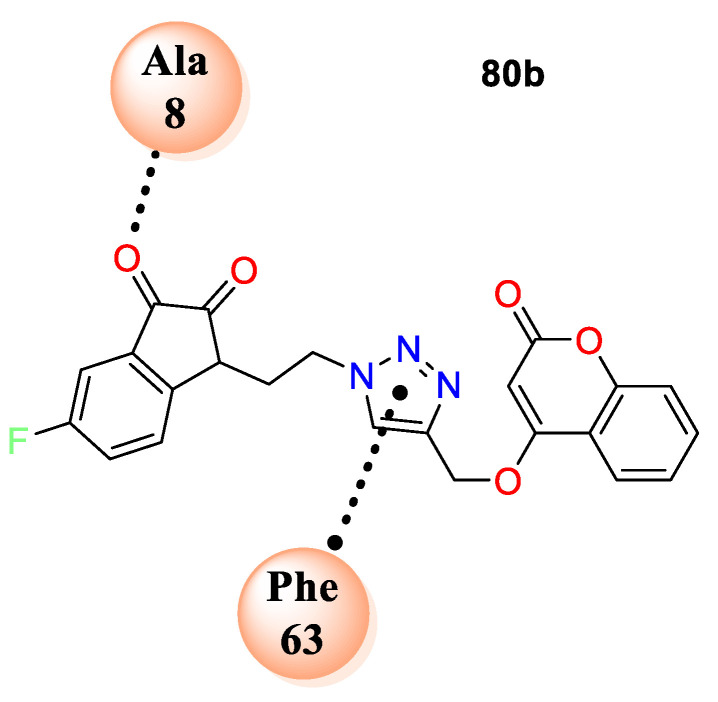
Docking conformation of **80b** at the active site of DHFR.

Kavita Bhagat et al. [26] synthesized the novel indolinedione−coumarin hybrids (Scheme 10) and screened for their antimicrobial potential against Gram-negative (*E. coli* and *Salmonella enterica*); Gram-positive (*S. aureus* and *Mycobacterium smegmatis*) bacterial strains; and four fungal strains (*C. albicans*, *Alternaria mali*, *Penicillium* sp., and *Fusarium oxysporum*) by using the agar gel diffusion method by Different substituted indolinedione (**76**) on treatment with 1,2-dibromoalkanes by using K_2_CO_3_ as a base in dimethylformamide to give **77** which on reaction with NaN_3_ in DMF at RT furnish 1-(4-azidoalkyl)indoline-2,3-diones (**78**). Intermediate 4(prop-2-ynyloxy)-2H-chromen-2-one (**79**) was obtained by reacting 4- hydroxycoumarin (**58**) and propargyl bromide under basic condition using K_2_CO_3_. Finally, the obtained intermediate on reaction with different substituted 1-(4-azidoalkyl) indoline-2,3-diones (**78**) in the presence of catalytic amount of pentahydrate CuSO_4_ and sodium ascorbate yields desired indolinedione−coumarin hybrids **80a–u**. From this series, analogue **80b** exhibited potent antibacterial activity against *S. aureus* and *S. enterica* with zone of inhibition 2.5 and 1.3 cm, respectively. Along with most of the hybrids have shown good potential against fungi *Penicillium* sp. analogue **80a** was found to exhibit excellent antifungal potential with zone of inhibition 2.5 cm. Similarly, **80b** was found to be the second promising hybrid with zone of inhibition1.3 cm against *Penicillium* sp. SAR analysis revealed that electron density at the fifth position of indolinedione framework greatly impacted antibacterial potential. The screening clarifies that chain length of two carbon atom which links the triazole moiety to indolinedione framework was the most tolerable linker while the unsubstituted indolindione is the highest crucial for antifungal potential. The docking study of the most active analogues from the series, i.e., **80b**, was accomplished on *S. aureus* DHFR (PDB ID: 3SRQ). Study demonstrates that compound **80b** fits well in the cavity of enzyme and gets stabilized by different electrostatic interactions including major van der Waal’s, π−π stacking, and H-bond interactions (Figure 6).

**Scheme 11:**
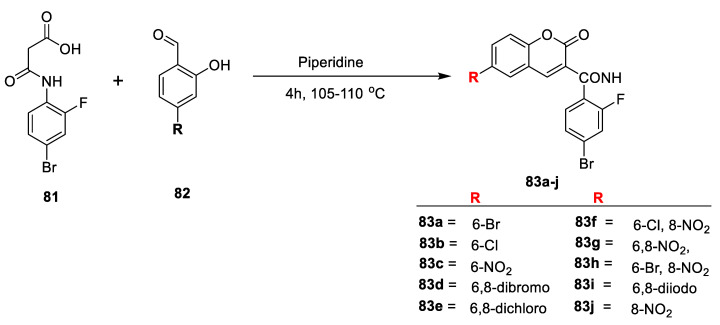
Synthetic scheme for coumarin analogues (**83a–j**). Reproduced and adapted with permission from Ref. [27]. under Creative Commons CC BY-NC-ND 4.0. Copyright 2019, Elsevier.

Mohd. Shahnawaz Khan et al. [27] reported an eco-friendly itinerary series of newly substituted chromene-3-carboxamide derivatives. The green synthesis approach involves condensation of substituted salicylaldehyde (**82**) with N-(substituted) phenyl malonic acid (**81**) in the presence of a base catalyst, piperidine obtained chromene-3-carboxamide derivatives **83a–j** (Scheme 11). The newly furnished derivatives were evaluated for their antimicrobial potential against two bacterial strains, *E. coli* (Gram-negative) and *B. cereus* (Gram-positive) and seven fungal strains, namely, *A. niger*, *A. fumigatus*, *A. flavus*, *Rhizopus*, *Mucor*, *Penicillium*, and *C. albicans*, by the agar well diffusion method using fluconazole as a reference drug. Compounds **83c**, **83d**, **83e**, **83f**, and **83j** exhibited broad-spectrum antibacterial potential due to the presence of bromo, chloro or nitro substitutions at C6 and C8 position of chromene ring l. The inhibitory potential against *E. coli* and *B. cereus* at 1000 μg/mL concentration with a zone of inhibition of 10–16 mm was reported. All the analogues from the series were also evaluated for their antifungal potential against seven fungal strains. Among them **83a** has shown moderate antifungal activity with MIC 500 μg/mL against *A. flavus*, *A. niger*, and penicillium, while **83b** was shown against *A. niger* and *C. albicans* with MIC of 500 and 250 μg/mL, respectively. Moreover, compounds **83c** and **83f** displayed lower MIC value against *A. fumigatus*, *A. flavus*, Mucor and Penicillium. Results conclude that compounds **83c–g** has displayed good to moderate antifungal potency against all tested fungal strains. Some analogues **83d** and **83e** exhibited excellent MIC of 125 μg/mL. 

**Scheme 12:**
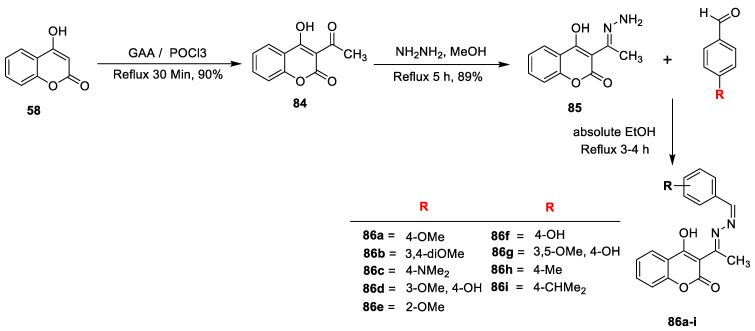
Synthesis of unsymmetrical azines (**86a–i**). Reproduced and adapted with permission from Ref. [28]. Copyright 2019, John Wiley and Sons.

Milenko N. Ristić and co-workers [28] carried out synthesis of some novel asymmetric azines containing coumarin analogues. The title hybrids were prepared using the synthetic strategy depicted in (Scheme 12). The coumarin ketone **84** obtained via acylation of **58** with add full form (GAA) in the presence of POCl_3_. Hydrazone of 4-hydroxy-3-acteyl coumarin **85** can be afforded by reaction of **84** with hydrazine hydrate in a 1:1 ratio using ethanol. The title compounds asymmetric azines (**86a–i**) were synthesized by reacting **85** with different substituted aromatic aldehydes in absolute ethanol. All the title compounds tested for antimicrobial potential against four Gram-positive, three Gram-negative bacterial strains and two fungal strains. Found results shows that all the compounds were effective inhibitors of microbial growth against all the tested strains except **86i** which is active against *C. albicans*. Compounds **86a** and **86f** displayed highest antimicrobial potency with MIC value of 1.32 and 1.4 µmol mL^−1^, respectively. Among the series compound **86c** found to be potent antifungal candidate. 

**Scheme 13:**
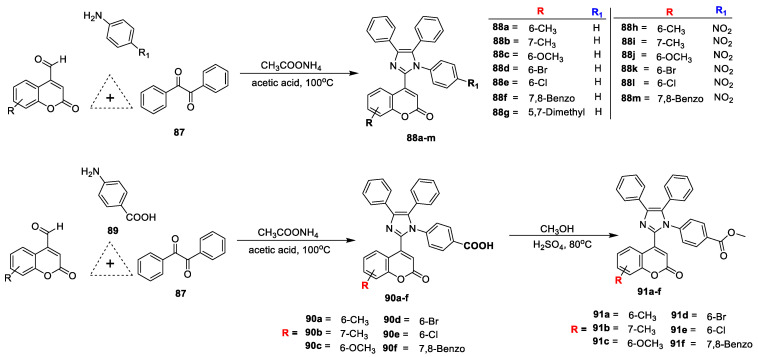
Outline for coumarin—imidazole conjugates **88a–m**, **90a–f** and **91a–f**. Adapted from Ref. [29].

Megharaja Holiyachi et al. [29] reported one-pot multi-component synthesis of tri and tetra-substituted coumarin-imidazole hybrids. Synthesized derivatives were tested for their antimicrobial potential against *Bacillus flexus* (Gram-positive) *Pseudomonas* spp. (Gram-negative) bacterial and *Scopulariopsis* spp. and *Aspergillus terreus* fungi strains using via the well diffusion method using ciprofloxacin and nystatin as reference drugs. Compounds were prepared as described in (Scheme 13). Reaction of mixture of 4-formylcoumarins, substituted anilines and 1, 2-diketone (**87**) with ammonium citrate under reflux condition in the presence of acid catalyst has yielded title analogues **88a–m**. Likewise analogues **90a–f** were obtained using the similar above-mentioned reaction conditions. Further corresponding ester **91a–f** were furnished by treatment of **90a–f** to sulphuric acid in methanol under reflux. Derived results have shown that most of the analogous display promising activity against both bacterial strains. Analogues having *N*-1-phenyl substation on imidazole and substitutions on coumarin ring **88a–g** possess excellent antibacterial potency with MIC values of 0.1 to 0.4 µg/mL except compound **88b**, while analogues bearing nitrobenzene substitution on N-1 imidazoles **88h–m** displayed higher activity. Compounds **91a–f** were also found to be promising inhibitors against both tested strains of bacteria at low concentrations. All synthesized candidates were screened for antifungal potency and found excellent inhibitors of *Scopulariopsis* spp. and *Aspergillus terreus* fungi strains. These antifungal results are almost like that of the antibacterial potential. SAR studies revealed from the series **88a–g** that compound **88b** with (R1-CH_3_ and R-H) at *N*-1-phenyl substitution was inactive, however introduction of electron-withdrawing species in **88i** (NO_2_), **90b** (COOH), and **91b** (COOCH_3_) on the *N*-1-phenyl ring exhibited potent antibacterial and antifungal activity. It indicates that for antimicrobial potential C7 substitution at coumarin nucleus and electron-withdrawing groups on *N*-1-phenyl ring at *p*-position is necessary. 

**Scheme 14:**
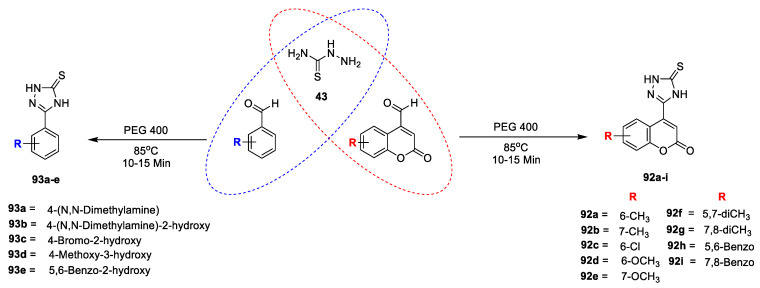
Outline for synthesis of substituted coumarin and phenyl-1,2,4-triazolidine-3-thiones (**92a–i**) and (**93a–e**). Adapted from Ref. [30]. Copyright 2019, John Wiley and Sons.

**Figure 7:**
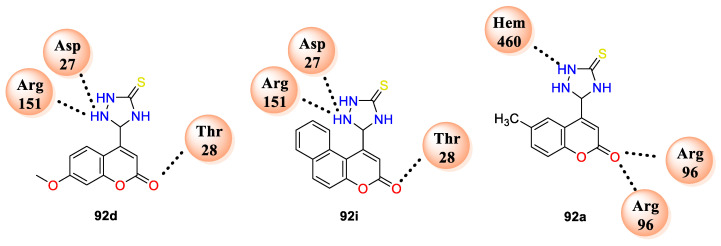
HB Interactions of compounds **92d** and **92i** with FabH target protein (PDB ID: 1HNJ) and **92a** with CYP51 (PDB ID: 1EA1).

Farzanabi Shaikh et al. [30] carried out the synthesis and evaluation of substituted coumarin and phenyl-1,2,4-triazolidine-3-thiones for their antitubercular and antimicrobial activity. Title analogues were prepared by two component rection of coumarin, phenyl and thiosemicarbazide using PEG-400. Initially intermediate thiosemicarbazone were obtained via nucleophilic addition of thiosemicarbazide (**43**) to the carbonyl carbon of substituted 4-formylcoumarine/benzaldehyde, followed by intramolecular nucleophilic attack of –NH_2_ of thiosemicarbazone to azomethine carbon to furnish title analogues coumarin and phenyl 1,2,4-triazolidine-3-thion **92a–i** and **93a–e** (Scheme 14). The title compounds were tested for their antibacterial potential against Gram-positive (*S. aureus* and *B. subtilis*) and Gram-negative (*E. coli* and *P. aeruginase*) and four fungal strains, namely, *C. albicans*, *A. niger*, *A. flavus*, and *A. fumigate by* using broth dilution method. From the obtained results it clears that all compounds were active against both Gram-positive and Gram-negative bacterial strains, as well as all tested fungi. Amongst the coumarin-fused triazolothiones (**92a–i**) series compounds **92a**, **92b**, **92c**, **92d**, and **92i** found to be highly potent against *S. aureus* and *Bacillus* spp., as well as *E. coli*, with MIC values ranging from 0.8 to 1.6 μg/mL while, standard ciprofloxacin exhibited MIC 2 μg/mL, whereas the in vitro antifungal evaluation concludes that all the hybrids displayed outstanding potential against fungal strains with MIC values ranging from 0.4 to 6.25 μg/mL. Molecular docking was performed against the *E. coli* FabH target protein (PDB ID: 1HNJ) to support given results. Compound **92d** makes three hydrogen bonding interactions with amino acids residues THR28, ASP27, ARG151, and compound **92i** makes interaction with amino acids THR28, ASP27, and ARG151, while compound **92a** against active site of enzyme cytochrome P450 14 alpha-sterol demethylase (CYP51; PDB ID: 1EA1) makes hydrogen bonding interaction with residues ARG96 and HEM460, as shown in (Figure 7). 

**Scheme 15:**
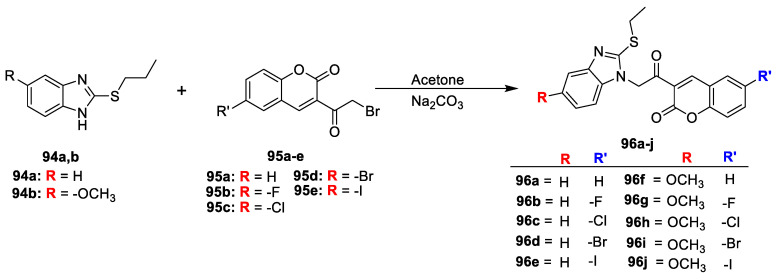
Development of butylthio—1H—benzimidazole-based coumarin derivatives **96a–j**. Adapted from Ref. [31]. under Creative Commons CC BY-NC-ND 4.0. Copyright 2019.

Mohd Imran [31] synthesized benzimidazole-based coumarins derivative for their antimicrobial and antioxidant activity. A mixture of substituted 2-(propylthio)-1H-benzo[d]imidazole (**94a,b**) and substituted 3-(2-bromoacetyl)-2H-chromen-2-one (**95a–e**) was stirred in acetone furnished title analogues 2-butylthio-1H-benzimidazole-based coumarin derivatives (**96a–j**) outlined in Scheme 15. All the synthesized compounds were screened for their antibacterial potential against *S. aureus* (ATCC-25923), *E. coli* (ATCC-25922), *E. faecalis* (ATCC-29212), *K. pneumoniae* (ATCC700603), *C. albicans* (ATCC-2091), and *P. citrinum* (NCIM-768). Compounds having –F, -Cl, and –Br groups at C-6 of the coumarin moiety together with –OCH_3_ at C-5 of the benzimidazole found to be potent against tested microorganism. 

**Scheme 16:**
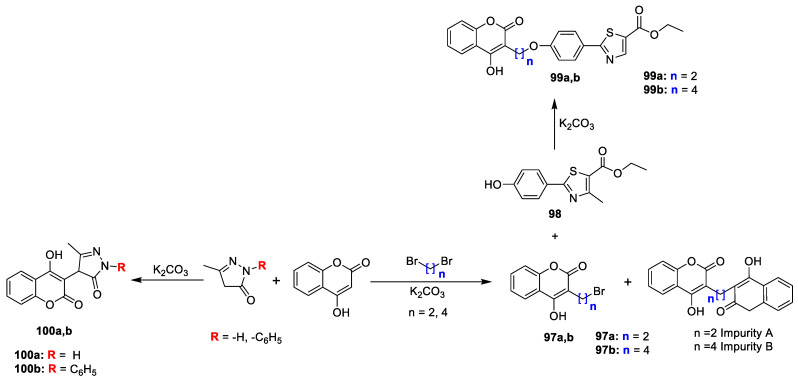
Synthesis of coumarin fused pyrazolones and methyl thiazole derivatives. Adapted from Ref. [32]. under Creative Commons CC BY-NC 4.0. Copyright 2019.

Chirag G. Naik et al. [32] worked on the synthesis of some novel coumarin fused pyrazolones and methyl thiazole derivatives for their antimicrobial potential. 3-(2-bromoethyl)-4-hydroxy-2*H*-chromen-2-one (**97a,b**) were obtained from 4-hydroxy coumarin and dibromo ethane in the presence of base. Synthesis of **99a,b** was done by reacting corresponding **97a,b** with ethyl-4,5-dihydro-2-(4-hydroxyphenyl)-4-methylthiazole-5-carboxylate (**98**) in acetone. Analogues **100a** and **100b** were prepared by reaction of 4-hydroxy coumarin with 3-methyl-1- phenyl-1H-pyrazol-5(4H)-one and 3-methyl-1H-pyrazol-5(4H)-one in the presence of K_2_CO_3_ (Scheme 16). Synthesized derivatives were evaluated for antimicrobial potential against *S. aureus*, *Bacillus subtilis* (Gram-positive) and *E. coli*, *P. aeruginosa* (Gram-negative) by using agar diffusion method and fungi *C. albicans* (MTCC 227) using Sabouraud dextrose agar medium. Compounds **99a** and **99b** were found to possess significant inhibitory potential against *P. aeruginosa* and *E. coli*, while compounds **99b** and **100a** displayed good to moderate activity against *Bacillus subtilis*. All the compounds revealed moderate potency against *Candida albicans* (fungi). 

**Scheme 17:**
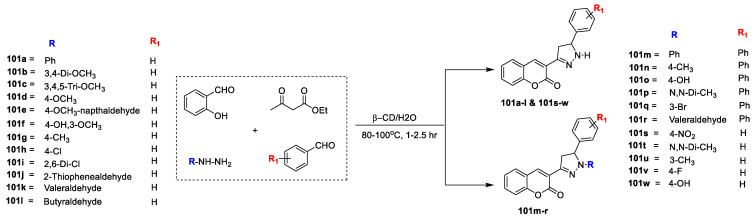
General scheme for the synthesis of **101a–w**. Adapted from Ref. [33].

Asha V. Chate and co-workers [33] reported a series of novel coumarin-linked pyrazoline inhibitors of D-alanine-D-alanine ligase via one-pot four component synthesis and screened for their antimicrobial potential against *E. coli* DdlB ligase. The outline for synthesis of title analogues is depicted in (Scheme 17). Title hybrids **101a–l** and **101s–w** and **101m–r** were obtained via one-pot four component synthesis between salicylaldehyde, ethyl acetoacetate, hydrazine hydrate, and benzaldehyde in water in the presence of cyclodextrin. Prepared analogues were subjected to in vitro evaluation for antibacterial and antifungal potential against human pathogenic bacterial strains (*E. coli*, *B. subtilis*, *S. aureus*) and fungal strains (*C. albicans*, *Candida glabrata*, *A. fumigates*, *A. flavus*, *A. niger* and *C. neoformans*) using Miconazole as a standard drug. In vitro antibacterial analysis revealed that compound **101f** is potent and has displayed excellent potential with MIC values of 14 µg mL^−1^, 14 µg mL^−1^ and 32 µg mL^−1^ against *E. coli* 1411, *E. coli* SM1411 and *S. aureus* NCIM-2901 respectively. The results are comparable to the standard drug D-cycloserine. Compound **101g** has proven to be the second best from the series with MIC of 16 µg mL^−1^, 18 µg mL^−1^ and 40 µg mL^−1^ against *E. coli* 1411, *E. coli* SM1411 and *S. aureus* NCIM-2901, respectively. Moreover, compounds **101i** and **101h** also displayed good potential among the series. Similarly, compound **101j** having thiophene moiety displayed excellent antifungal potential than the standard drug miconazole against *C. albicans*, *C. glabrata*, *F. oxysporum*, and *A. fumigates* with MIC values 20 µg mL^−1^, 20 µg mL^−1^, 22 µg mL^−1^, 34 µg mL^−1^, 12 µg mL^−1^, 14 µg mL^−1^ and 12 µg mL^−1^ respectively. The SAR analysis revealed that candidates **101m–r** with substitution on ‘NH’ group of pyrazole moiety are inactive or displayed less antibacterial potential as compared to the compounds having no substitution on ‘NH’ group of pyrazole moiety (**101a–l**). Candidates having di-chloro substitution on phenyl ring (**101i**) was found more active than compound **101h** with *p*-chloro substitution on the phenyl ring. Analogue **101c** bearing 3,4,5-tri-methoxy substitution on the phenyl ring exhibited more antifungal potency than compound **101b** containing 3,4-dimethoxy substitution on the phenyl ring. Compound 3-(5-(3-methoxynaphthalen-2-yl)-4,5-dihydro-1Hpyrazol-3-yl)-2H-chromen-2-one) (**101e**) also found to possess good potential against tested fungi. 

**Scheme 18:**
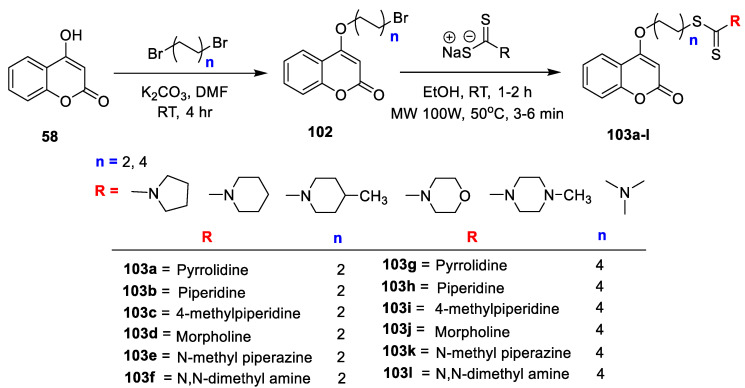
Design of coumarin bearing dithiocarbamate **103a–l**. Reproduced and adapted with permission from Ref. [34]. Copyright 2018, Elsevier.

**Figure 8:**
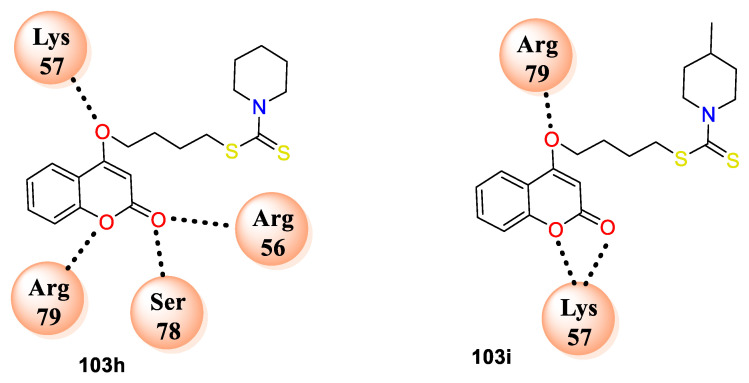
Hydrogen bonding interactions of compounds **103h** and **103i** with *C. albicans* DHFR (PDB ID: 1Al9).

Coumarin bearing dithiocarbamate derivatives were synthesized and evaluated by their in vitro antimicrobial potential against both Gram-positive, Gram-negative bacterial strains by S.N. Mangasuli et al. [34]. The study includes evaluation against bacterial strains, namely, *S. aureus*, *B. subtilis*, *E. coli*, and *P. aeruginosa*, and fungal strains, namely, *A. flavus*, *Trichoderma harzianum*, and *Penicillium chrysogenum*, and yeast *C. albicans* using the macro-dilution broth method. Bromoalkoxy-chromen-2-ones (**102**) were obtained by treatment of aliphatic dibromo species with **58** in the presence of K_2_CO_3_ in DMF. Condensation of 4-(2-bromoethoxy)-2Hchromen-2-one with dithiocarbamate salt in ethanol furnished desired 2-(2-oxo-2H-chromen-4-yloxy) ethyl pyrrolidine1-carbodithioate analogues **103a–l** (Scheme 18). Compound **103i** was found to be the most potent from the series with MIC of 0.5 mg/mL against *S. aureus*, 1 mg/mL against *B. subtilis*, 2 mg/mL against *E. coli* and *P. aeruginosa*. Likewise, compound **103h** displayed good potential against *S. aureus*, *B. subtilis* and *P. aeruginosa* with MIC of 1 mg/mL and against *E. coli* with MIC of 1 mg/mL. Further, most of the compounds from the series have shown good to moderate antibacterial activity as compared to the standard. Moreover, prepared candidates were screened for their antifungal potential. Among them compound **103i** displayed excellent potential against *A. flavus*, *T. harzianum* and *C. albicans* with MIC 1 mg/mL and *C. albicans* with 0.5 mg/mL which is very less MIC value as compared to the standard. Similarly, compound **103h** exhibited activity against *A. flavus*, *T. harzianum* and *C. albicans* with MIC of 1 mg/mL and 0.5 mg/mL against *P. chrysogenum*. SAR studies explored that candidate **103i** with electron-donating species (-CH_3_) at *p*-position of piperidine framework of dithiocarbamate linked via alkyl ether linkage -O(CH_2_) nS- (*n* = 4) to coumarin scaffold displayed lower MIC 0.5 mg/mL as compared to the standard analogue. Likewise, the analogue **103i** has shown a C-score value of 7.57 which is superior in comparison to the standard fluconazole. SAR also revealed variation in alkyl chain of the respective derivatives (*n* = 4) with different substituents found excellent in activity in comparison with alkyl chain of *n* = 2. Docking results have shown that all the docked analogues exhibited good docking score against *C. albicans* DHFR (PDB ID: 1Al9). Active compounds from series **103h** makes hydrogen bonding interactions with amino acid residues ARG56, SER78, ARG79, LYS57 and **103i** with ARG79, LYS57 (Figure 8). The study has proposed mechanism of action for coumarin bearing dithiocarbamate analogues by inhibiting the dihydrofolate reductase enzyme.

**Scheme 19:**
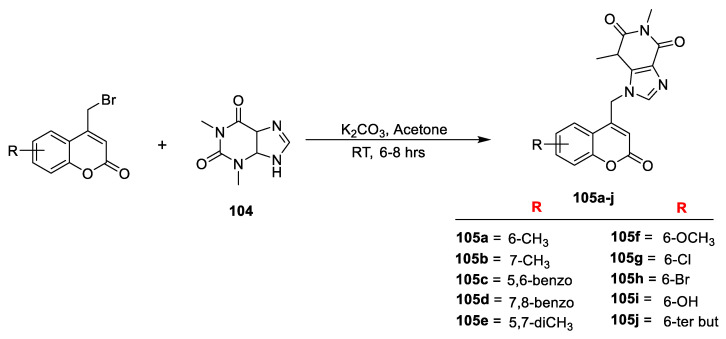
Development of coumarin—theophylline hybrids **105a–j**. Reproduced and adapted with permission from Ref. [35]. Copyright 2018, Elsevier.

In another study S. N. Mangasuli and co-workers [35] contributed to synthesis of novel coumarin-theophylline hybrids and screened their antitubercular and antimicrobial potential. The synthesis methodology is outlined in (Scheme 19) were starting from substituted 4-bromomethyl coumarin were obtained via Pechmann cyclization of phenol with 4-bromoethylacetoacetate in sulphuric acid. The furnished substituted 4-bromomethyl coumarins upon condensation with theophylline (**104**) in the presence of anhydrous K_2_CO_3_ in acetone yield 1,3-dimethyl-9-[(substituted-2-oxo-2Hchromen-4-yl) methyl)-1H-purine-2,6(3H,9H]-dione derivatives (**105a–j**). The prepared derivatives were screened for their in vitro antimicrobial potency against *S. aureus* (Gram-positive) and *E. coli*, and *S. typhi* (Gram-negative) bacteria, as well as fungi *C. albicans.* Among the series, compound **105a** with 6-CH_3_ substitution was found active against *S. aureus*, *E. coli* with MIC of 3.9 µg/mL and against *S. typhi* with MIC of 7.8 µg/mL. Additionally, compound **105c** with 5,6-Benzo, **105f** with 6-OCH_3_, and **105j** with 6-tert-butyl substitutions displayed good antibacterial potency against *S. aureus* and for *E. Coli* with MIC of 7.8 µg/mL, 7.8 µg/mL, and 3.9 µg/mL, respectively. The antiviral potency was compared with Amphotericin B as the standard drug. From the obtained results, compounds **105a** (6-CH_3_), **105b** (7-CH_3_), and **105f** (6-OCH_3_) were found most active against *C. albicans* with a MIC value of 31.3 µg/mL. Additionally, **105c**, **105d**, **105i**, and **105j** displayed sensible potential against *C. albicans* with MIC value of 62.5 µg/mL. The SAR studies have shown that presence of electron-donating species substituted to C-6 of coumarin moiety contributes towards the antibacterial potential.

**Scheme 20:**
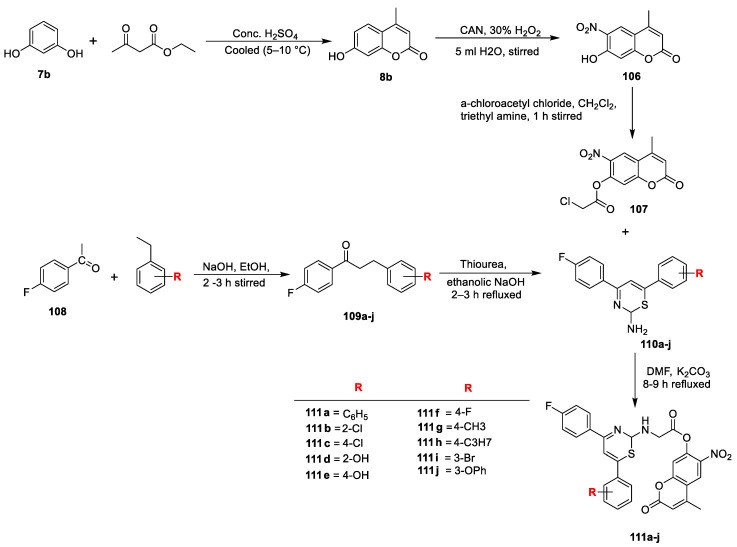
Scheme for coumarin clubbed thiazine scaffolds **111a–j**. Reproduced and adapted with permission from Ref. [36]. Copyright 2018, Springer Nature.

Nilesh B. Chauhan et al. [36] reported a new series of 4-methyl-6-nitro-2-oxo-2*H*-chroman-7yl-2-(4-(4-fluorophenyl)-6-phenyl-2*H*-1,3-thiazin-2-yl-amino) acetates (Scheme 20). Compound **8b** was synthesized via Pechmann condensation, which, on nitration with nitric acid, obtains **106**, followed by a reaction with chloroacetyl chloride, gives 4-methyl-3-nitro-2-oxo2H-chromen-7-yl 2-chloroacetate (**107**). Title derivatives **111a–j** were obtained by condensation of **107** with amino thiazine derivatives (**110a–j**) via cycloaddition reaction in between substituted chalcones (**109a–j**) and thiourea. The test compounds were screened for their antimicrobial potential against four bacterial and three fungal strains using broth microdilution method and compared with chloramphenicol, ciprofloxacin, and griseofulvin as a standard. Analogue **111c** attached to -Cl and **111h** attached to –CH_2_CH_2_CH_3_ at C-4 of benzaldehyde were found to be more active against *E. coli* with MIC value of 50 µg/mL and the results were compared to standard drugs chloramphenicol and ciprofloxacin, whereas compounds **111a** (-H) and **111b** (2-Cl) displayed significant antifungal potential against *S. pyogenes* with MIC value of 250 µg/mL compared with griseofulvin. Other test analogues were found to be less to moderately active. 

**Scheme 21:**
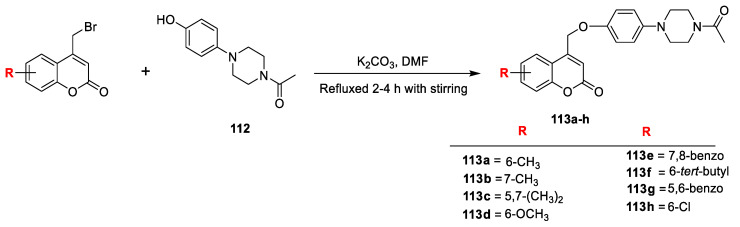
Schematic representation of coumarin-piperazine derivatives. Reproduced and adapted with permission from Ref. [37]. Copyright 2018, Elsevier.

Synthesis of some coumarin-piperazine derivatives was carried out by Shrinivas Koparde et al. [37]. Outline for synthesis of title compounds is depicted in (Scheme 21). Substituted 4-bromomethyl coumarins were obtained via Pechmann cyclization of phenols with 4-bromoethylacetoacetate. Title coumarin-piperazine derivatives (**113a–h**) were prepared by condensation of obtained substituted 4-bromomethyl coumarins with 1-(4-(4-hydroxyphenyl) piperazin-1-yl) ethanone (**112**) in the presence of K_2_CO_3_ using DMF as solvent. Label hybrids evaluated for their in vitro antimicrobial activity against both Gram-negative and Gram-negative bacterial strains, as well as four fungi. Among the series compound **113a** was exhibited excellent potential against *S. aureus* and *E. faecal* (MIC = 0.5 µg/mL) and with *E. coli* and *P. aeruginosa* with MIC value 1 µg/mL, whereas **113d** and **113f** were found as good in action against *S. aureus* and *E. faecal* with MIC value 2 µg/mL. Antifungal potential of the test compounds was evaluated against *A. flavus*, *P. chrysogenum*, *Trichoderma harzianum*, and *C. albicans*. Obtained results have concluded that displayed compound **113a** has excellent activity against *A. flavus* and *T. harzianum* with MIC of 0.5 µg/mL and against *P. chrysogenum* and *C. albicans* with MIC value of 1 µg/mL, which is equivalent to the standard fluconazole. Moreover, compounds **113d**, **113f**, and **113h** exhibited good potency with MIC 2 µg/mL against *A. flavus*, *T. harzianum* and *P. chrysogenum* when compared to the standard. Further, molecular docking studies were encouraged and supported the results of in vitro antimicrobial activities, the compounds **113a**, **113e**, **113f**, and **113h** have higher C score values than that of the standard drug ciprofloxacin. 

**Scheme 22:**
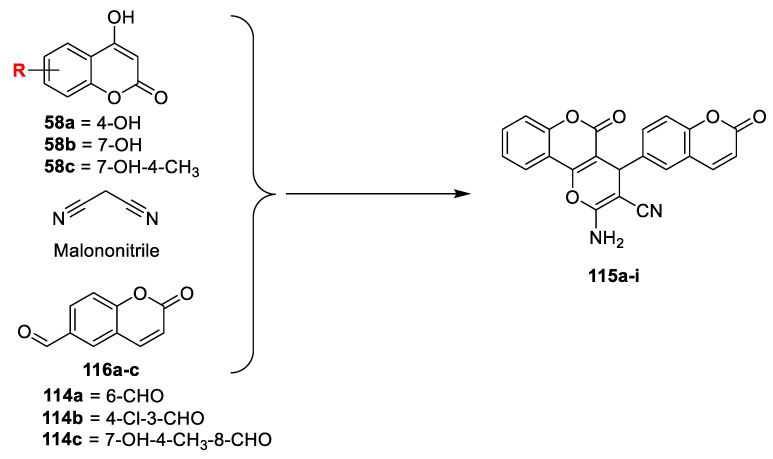
Schematic representation of biscoumarin derivatives fused with dihydropyran ring. Reproduced and adapted with permission from Ref. [38]. Copyright 2018, John Wiley and Sons.

V.K.A. Kalalbandi et al. [38] have illustrated in vitro antimicrobial potency of some dihydropyran-bis coumarins. 6-formylcoumarins (**114a**) were synthesized via the Reimer−Tiemann reaction in the presence of chloroform and KOH. 3-formyl-4-cholorocoumarin (**114b**) furnished through the Vilsmeier−Haack reaction of 4-hydroxy coumarin in POCl_3_-DMF and 8-formyl-7-hydroxy-4-methylcoumarin (**114c**) prepared via Duff reaction of 7-hydroxy-4-methylcoumarin. Title compounds dihydropyran-bis coumarins (**115a–i**) were synthesized via one pot multicomponent reaction of different substituted hydroxycoumarins, formyl coumarins (**114a–c**) and malononitrile in the presence of catalytic amount of triethylamine in methanol (Scheme 22). The in vitro antimicrobial activity was carried out against bacterial strains, namely, *E. faecalis*, *S. aureus*, *P. aeruginosa*, and *E. coli*, and fungi *C. albicans* and *A. Niger* using broth micro dilution method. Biological study exposes most of the derivative active against Gram-positive bacterial strains and compounds **115a** and **115b** have emerged as a most potent form the synthesized hybrids by exhibiting MIC of 0.4 and 0.8 mg mL^−1^, respectively, against *S. aureus* as compared to the standard ciprofloxacin. Additionally, **115a** was found active against Gram-negative strain *P. Aeruginosa*, while the rest of the test compounds were moderately active against remaining microorganisms. On the other hand, compounds **115a** and **115b** also found significantly active against *C. albicans* with MIC values 0.8 and 0.4 mg mL^−1^, respectively. The SAR study concluded that core biscoumarin attached dihydropyran moiety with no substitution found better in potency against Gram-positive strains as compared to the substitution on core biscoumarin attached dihydropyran framework.

**Scheme 23:**
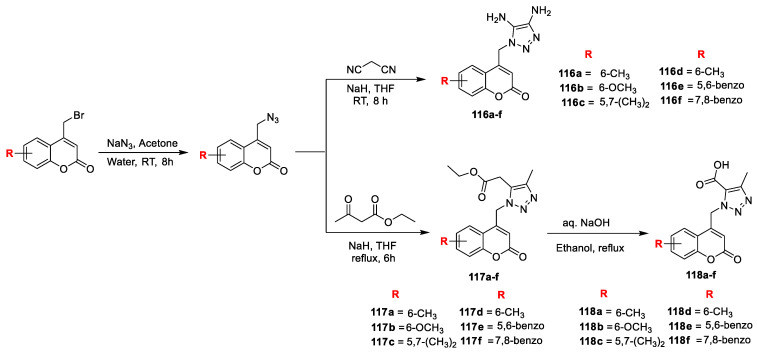
Schematic representation of 4–substituted coumarino-1,2,3-triazole. Reproduced and adapted with permission from Ref. [39]. Copyright 2018, Elsevier.

Jyoti M. Madar and co-workers [39] developed a new series of structurally identical coumarin triazoles and evaluated for their cytotoxic and antimicrobial action. Initially, substituted 4-bromomethyl coumarins obtained via Pechmann cyclization which is converted into substituted dipolar coumarin azides by reaction with sodium azide in acetone. 1,4,5-trisubstituted-1,2,3- triazoles (**116a–f**) were prepared via base catalysed cycloaddition reaction of substituted dipolar coumarin azides with malononitrile (active methylene compounds). In the same way, coumarin triazole carboxylate (**117a–f**) were obtained by the reaction of substituted coumarin azide with ethylacetoacetate in the presence of NaH in THF via reflux. The yielded compounds **117a–f** were converted to coumarin triazole carboxylic acid (**118a–f**) using NaOH under reflux (Scheme 23). The newly furnished analogues **116a–f**, **117a–f** and **118a–f** were screened for their in vitro antimicrobial activity against different bacterial and fungal strains. Out of the tested hybrids from the series of coumarin triazole amino carbonitriles (**116a–f**), compounds **116a**, **116b**, **116d**, and **116e** were proved as excellent inhibitors against *S. aureus* with MIC values 0.4, 0.8, 0.4, and 0.4 μg/mL, respectively, when compared to the standard Ciprofloxacin with MIC value 2 μg/mL. In addition, **116a**, **116c** and **116e** displayed good potential against *E. coli* with MIC value 3.12, 12.5 and 1.6μg/mL, respectively. From the series of coumarin triazole carboxylate (**117a–f**), compounds **117d**, **117e**, and **117f** exhibited excellent potency against *S. aureus* with MIC values 0.8, 0.8 and 0.4 μg/mL, respectively, when compound **117b** and **117e** displayed good potential against *E. coli* with MIC 3.12 and 12.5 μg/mL when compared to the standard. Furthermore, from the series **118a–f**, three analogues **118d**, **118e**, and **118f** found excellent activity against *S. aureus* with MIC values 0.2, 0.8 and 0.4μg/mL, respectively. Wherein candidate **118d** has proven to be good in activity against *E. coli*. The antifungal screening has revealed that among the 18 furnished candidates,**116a**, **116b**, **116d**, **116e**, **117f**, and **118e** emerging as excellent candidate with MIC values 0.4, 12.5, 3.12, 1.6, 6.25, and 6.25 μg/mL, respectively, against *A. niger* compared to the standard fluconazole. SAR study revealed that electron-releasing groups (CH_3_ and OCH_3_) at C5, C6 and C7 of coumarin moiety contributes to the antibacterial potency of the title compounds.

**Scheme 24:**
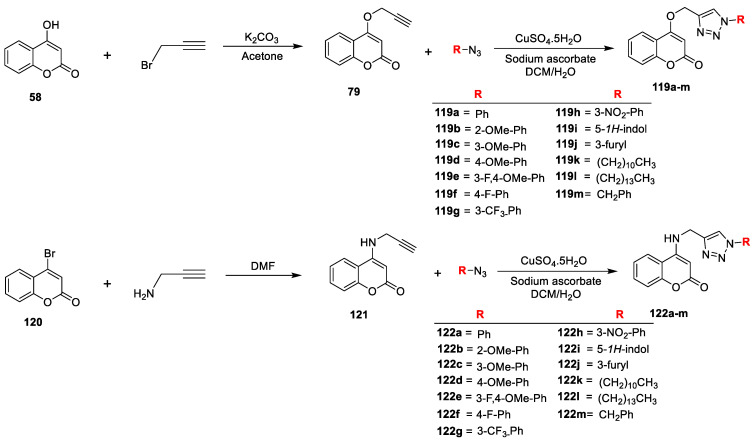
Outline for the synthesis of 4–substituted 1,2,3—triazole—coumarin. Adapted from Ref. [40]. under Creative Commons CC BY 4.0. Copyright 2018, MDPI.

Priscila López-Rojas et al. [40] analysed in vitro antimicrobial activity of novel series of 4-substituted 1,2,3-triazole-coumarin derivatives. The synthesis of title analogues is depicted in (Scheme 24). When 4-hydroxy-coumarin (**58**) reacted with 3-bromoprop-1-yne in the presence K_2_CO_3_ in anhydrous acetone it gives O-propargylated coumarin (**79**). Similarly, 4-bromo-coumarin (**120**) on reaction with prop-2-yn-1-amine via nucleophilic substitution reaction in DMF afforded N-propargylated coumarin (**121**). The title 4-substituted 1,2,3-triazole-coumarin derivatives (**119a–m** and **122a–m**) prepared via copper(I)-catalyzed Huisgen 1,3-dipolar cycloaddition reaction of corresponding O-propargylated coumarin (**79**) and N-propargylated coumarin (**121**) with different substituted alkyl or aryl azides, respectively. The synthesized analogous were tested against *S. aureus* (ATCC 6538), *E. faecalis* (PCM 2673), *E. coli* (ATCC 8739), *K. pneumoniae* and yeast *C. albicans* (ATCC 10231) and were compared with the standard chloramphenicol and ketoconazole. Among the screened series, compounds **119a**, **119b**, **119f**, **122h** and **122k** have exhibited promising activity with MIC ranging from 12.5 to 50.0 µg/mL against *E. faecalis*. Compound **119b** with 2-OMe–Ph group attached at the triazole moiety and an –OCH_2_– linker remained the finest candidate of the series. In the nitrogenated series candidate **122h** with 3-NO_2_–Ph and **122k** with an undecyl chain has proven to possess the best potential. 

**Scheme 25:**
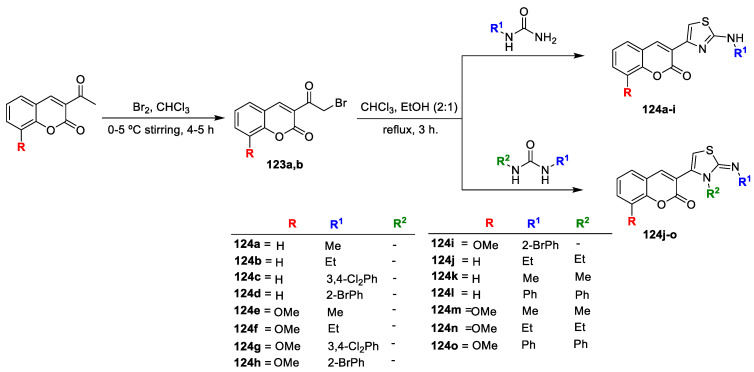
Scheme for the preparation of thiazolyl coumarin **124a–o**. Reproduced and adapted with permission from Ref. [41]. Copyright 2018, Elsevier.

Antibacterial, antitubercular, and antiviral activity of a new thiazolyl-coumarin hybrids was executed by Hasnah Osman and co-workers [41]. Initially, the substituted 3-acetylcoumarins on bromination with ethanol free chloroform afford key intermediates substituted-3-(2-bromoacetyl)-2H-chrome-2-ones (**123a,b**). Subsequently thiazolyl-coumarin analogues (**124a–o**) were obtained via Hantzsch cyclization of the corresponding intermediate **123a,b** with *N*-substituted and *N*,*N*-disubstituted thiourea (Scheme 25). Analogues were tested by in vitro antibacterial activity against two bacteria *S. pneumonia* and *S. aureus* (Gram-positive) and *E. coli*, *E. aerogenes* and *S. typhi* (Gram-negative) by using broth microdilution method. The obtained results were compared with the standard drugs streptomycin, kanamycin, and vancomycin. Out of the tested hybrids, compounds **124d**, **124h**, **124k**, and **124m** have exhibited promising antibacterial action. The bromophenol analogues **124d** (MIC 79 µM) and **124h** (MIC 73 µM) displayed highly significant inhibition against all the tested microorganisms with MIC values fairly less than that of vancomycin (MIC 86–176 µM). The disubstituted *N*,*N*-diphenyl candidate **124l** exhibited very low potency (MIC 158–316 µM). However, replacement with bulky alkyl groups led to compounds retaining their antibacterial potency such as **124k** (MIC 115–230 µM) and **124m** (MIC 49–98 µM). From the obtained results, the SAR analysis revealed that mono-substituted thiazoles remained more effective as compared to the di-substituted thiazoles. Candidates having lipophilic electron-withdrawing bromo group at the phenyl ring displayed promising potential while substitution of electron releasing (-OCH_3_) group at coumarin moiety is contributing towards increased potency. 

**Scheme 26:**
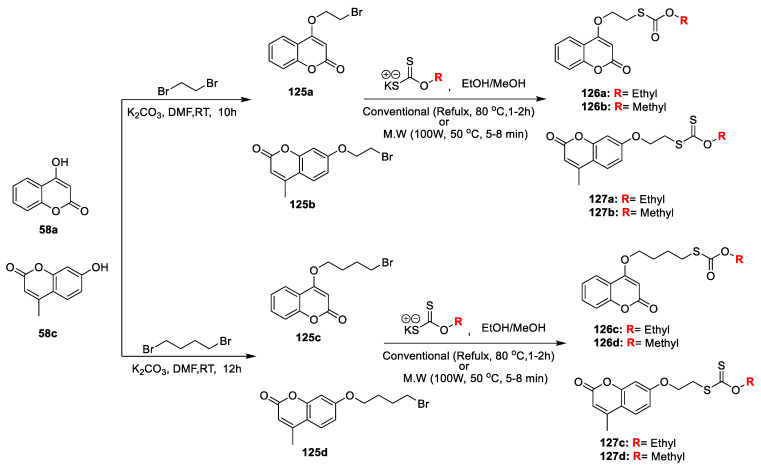
Development of coumarin—carbonodithioate hybrids. Reproduced and adapted with permission from Ref. [42]. Copyright 2018, Elsevier.

A new series of coumarin−carbonodithionate hybrids were contributed by Sumitra N. Mangasuli and co-workers [42] to evaluate their in vitro antimicrobial potential. The preparation strategy is as depicted in (Scheme 26). The mixture of 4-hydroxy (**58a**) and 7-hydroxy coumarins (**58c**) treated with various dibromoalkanes and anhydrous K_2_CO_3_ in DMF gives bromoalkoxy coumarins (**125a–d**). The obtained intermediates **125a–d** when condensed with potassium O-ethyl/methyl carbonodithioate in absolute ethanol furnished 4- and 7-substituted coumarin-carbonodithioate hybrids (**126a–d** and **127a–d**) via microwave irradiation technique. Prepared candidates were tested for their in vitro antimicrobial potential against *S. aureus*, *B. subtilis*, *E. coli* and *P. aeruginosa* and fungi *A. flavus*, *T. harzianum*, and *P. chrysogenum*, as well as yeast C. *albicans*.

Among the synthesized compounds, **126c** and **126a** have displayed excellent antibacterial potency against *S. aureus* and *B. subtilis* with MIC value 0.5 µg/mL and 2 µg/mL, respectively, and against *E. coli* and *P. aeruginosa* with MIC 1 µg/mL and 4 µg/mL, respectively. Moreover, analogues **126b** and **126d** have displayed good to moderate potential against tested pathogens. Compound **126c** also demonstrated potential against *A. flavus*, *T. harzianum*, and *P. chrysogenum* (MIC 0.25 µg/mL) and against *C. albicans* with (MIC 1 µg/mL), while candidate **126b** was found to possess significant activity against *A. flavus*, *T. harzianum*, and *P. chrysogenum* (MIC 0.5 µg/mL) and *C. albicans* with (MIC 1 µg/mL) as compared to the standard fluconazole, while the rest of the test compounds were found good to moderately active against tested fungal strains.

**Scheme 27:**
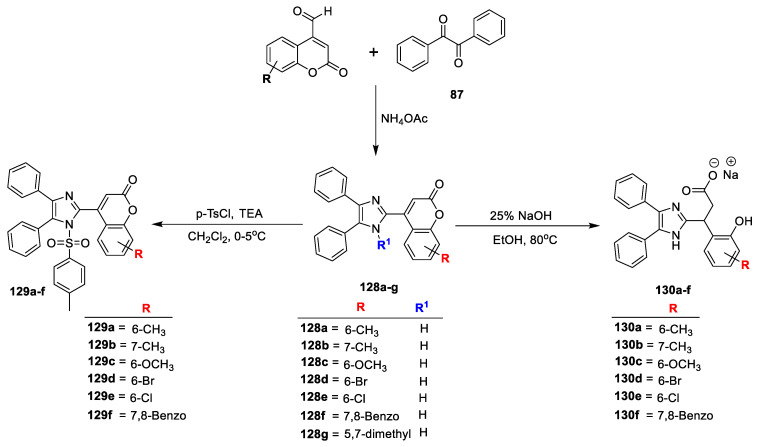
Synthesis of the coumarin–imidazole hybrid and phenyl imidazole acrylates. Reproduced and adapted with permission from Ref. [43]. Copyright 2018, Springer Nature.

Megharaja Holiyachi and co-workers [43] designed and synthesized by one pot multicomponent method some coumarin–imidazole analogues and phenyl imidazoloacrylates for both antimicrobial and anti-inflammatory potential. Initially, coumarin–imidazole hybrids (**128a–g**) were prepared by reacting 4-formycoumarin and 1,2-diketone (**87**) in the presence of the ammonium citrate as catalyst via conventional method. The next step obtained **128a–g** on treatment with *p*-toluenesulfonyl chloride under triethylamine and yields coumarin– imidazole sulphonamides (**129a–f**). On the other hand, analogues **128a–g** on treatment with sodium hydroxide via ring opening mechanism produced analogues **130a–f** which is isolated in the form of sodium salts (Scheme 27). The assay was performed against *Bacillus flexus* (Gram-positive) and *Pseudomonas* spp. (Gram-negative) bacterial strains, as well as fungi *Scopulariopsis* spp. and *Aspergillus terreus* via the agar well diffusion method using ciprofloxacin and nystatin as the reference candidates. The derived results conclude that all the screened analogues are active against both the tested bacterial strains compared to the standard drug. Compounds **128c** with –OCH_3_ and **128e** having –Cl at C-6 of coumarin displayed excellent potential with MIC value 0.3 μg/cm^3^. N-sulfonyl bearing imidazole derivatives (**129a–f**) has shown slight increase in the potential compared to compounds **128a–g**. Compound **129d** with bromo substitution at C6 position of coumarin and **129f** having 7,8-benzo substitution on the coumarin with MIC value 0.2 μg/cm^3^. The newly formed modified coumarin–imidazole hybrids to sodium salt of phenyl acrylate of imidazole **130a–f**, were found highly active against both tested bacterial strains compared to the standard and the other series of analogues **128a–g** and **129a–f**.

**Figure 9:**
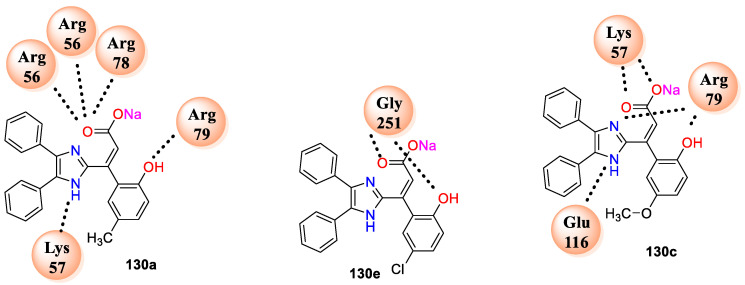
Hydrogen bonding interactions of compounds **130a** and **130c** with **1AI9** and **130c** with N-myristoyl transferase (PDB ID: 4CAW).

Among this series compound **130c** having –OCH_3_ and –OH on benzene, **130e** with chloro substitution and –OH on benzene ring and **130f** emerged as highly potent with MIC 0.1 μg/cm^3^. From the above evidence, analogues **130a–f** displayed excellent potential against both bacterial strains, and it might be the ionic nature of molecules which are completely soluble in water, while, against the tested fungal strains, analogues **128a**, **128b**, **128e**, and **128g** found active with MIC ranges from 1 to 4 μg/cm^3^. In case of series **129a–f**, all the complexes exhibited promising activity against both fungi except **129d** having bromo substitution at C6 position of coumarin, whereas improved activity was observed in case of series **130a–f** with MIC value ranging from 1 to 2 μg/cm^3^. Further, to explore antimicrobial potential of the synthesized analogues molecular docking study was perform against *C. albicans* dihydrofolate reductase (PDB IDs: 1AI9) and *A. fumigatus* N-myristoyl transferase (PDB ID: 4CAW; A Chain). Among the series compounds **130a–f** exhibited very good interaction with the selected enzyme target. Compound **130a** shows six hydrogen bonding interactions with cavity of enzyme 1AI9 involved amino acids residues LYS57 and ARG79 and three others with ARG56, while compound **130c** displayed hydrogen-bonding interactions with amino acids, namely, LYS57, ARG79, and GLU116, in the active pocket of the enzyme. Moreover, compound **130e** has shown interaction at the active site of N-myristoyl transferase (PDB: 4CAW; A-Chain with amino acids GLY25 and GLY251) (Figure 9). All the three analogues displayed better C score values against the enzyme (PDB ID: 1AI9). 

**Scheme 28:**
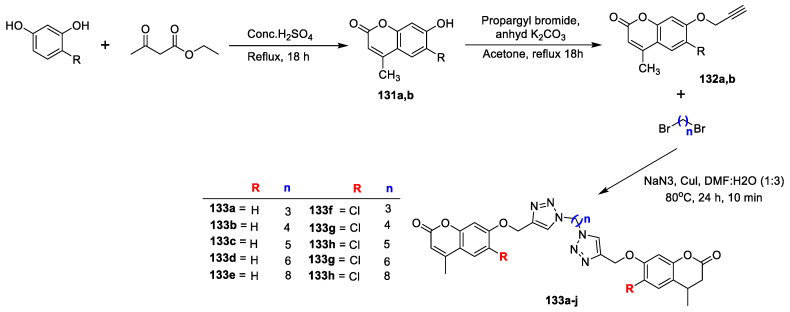
Synthetic route for the dimers of coumarin—tethered 1,2,3—triazole hybrids. Reproduced and adapted with permission from Ref. [44]. Copyright 2017, Elsevier.

Several dimers of coumarin-1,2,3-triazole hybrids bearing alkyl spacer were synthesized and evaluated for their antimycobacterial and antimicrobial potency by A. Dongamanti et al. [44]. An outline for the synthesis of new dimers is presented in (Scheme 28). 6-substituted-7-hydroxycoumarins (**131a,b**) were prepared via Pechmann condensation of resorcinols with ethyl acetoacetate. 6-substituted-4-methyl-7-(prop-2-yn-1-yloxy)-2H-chromen-2-ones (**132a,b**) were obtained by the reaction of the corresponding **131a,b** with propargyl bromide in the presence of potassium carbonate in acetone under reflux. Coumarin–1,2,3-triazole hybrids (**133a–j**) were afforded via nucleophilic substitution reaction of dibromoalkanes with sodium azides to produce in situ diazidoalkanes, which is coupled with **132a,b** by copper-catalyzed azide-alkyne 1,3-dipolar cycloaddition reaction. The in vitro antimicrobial screening was performed against *B. subtilis*, *S. aureus*, *E. coli* and *Proteus vulgaris* bacterial strains and *A. niger* and *C. albicans* fungal strains via Agar well-diffusion method using gentamicin and fluconazole as reference drugs.

Among the series compound **133j** was found to be highly active antibacterial candidate against *B. subtilis*, *S. aureus* and *E. coli* (MIC 3.125 µg/mL) with zone of inhibition 19, 16 and 14 mm, respectively. Additionally, compounds **133e** and **133i** were found as promising inhibitors against tested organism with MIC value of 6.25 µg/mL, while analogues **133d** and **133j** displayed good antifungal potential (MIC 12.5 µg/mL) with a zone of inhibition 12 and 14 mm against *A. niger* and 14 and 10 mm in contrast to *C. albicans*, respectively. Along with that, hybrids **133e** and **133i** exhibited good to moderate potential (MIC 12.5–25 µg/mL) with inhibitory zone of 12 mm, 16 mm and 10 mm, 11 mm, respectively. The SAR study proposed that compounds having lipophilic n-octyl and n-hexyl spacer and chloro substitution on the coumarin moiety were found to be promising for antimicrobial potential. 

**Scheme 29:**
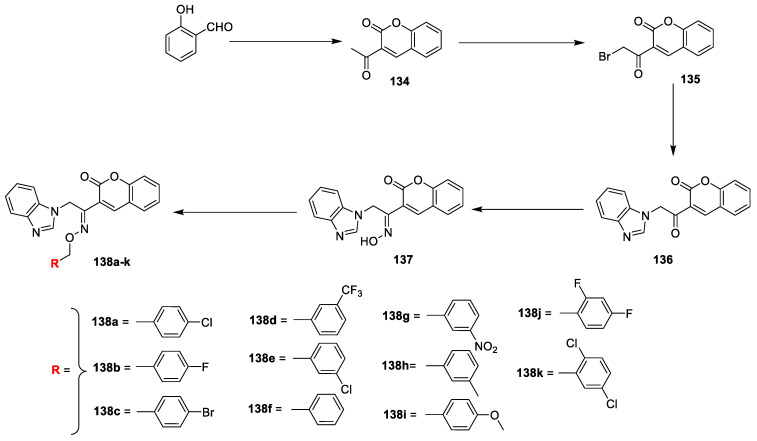
Synthesis of coumarin—benzimidazole conjugates. Reproduced and adapted with permission from Ref. [45]. Copyright 2017, Springer Nature.

LR Singh et al. [45] analysed in vitro antibacterial activity of coumarin-benzimidazole conjugates. The reported compounds were synthesized by a procedure depicted in (Scheme 29). The salicylaldehyde and ethyl acetoacetate in the presence of piperidine was converted into intermediate **134** via Knoevenagel condensation, which on bromination in dry chloroform yields compound **135**. The obtained intermediate **135** was reacted with benzimidazole in acetonitrile gave **136** via nucleophilic substitution reaction. Later condensation of **136** with hydroxyl amine hydrogen chloride under refluxed furnish key intermediate **137**. Finally, the title analogues **138a–k** were achieved by the reaction of **137** with different substituted benzyl halides under potassium tert-butoxide in DMSO via etherification. The synthesized derivatives were screened against *B. subtilis*, *S. aureus* (Gram-positive) and *P. aeruginosa*, *Proteus vulgaris*, *E. coli* (Gram-negative) bacterial strains. MIC values were established by using ampicillin, kanamycin, tetracycline, and ciprofloxacin as standard drugs. The derived biological data represents, compounds **138a** and **138c** have efficiently inhibited growth against the *B. subtilis* with MIC values 0.95 and 6.25 μg ml^−1^, respectively, while compounds **138c**, **138f**, **138j** and **138k** exhibited better potential against *P. vulgaris* with MIC values 1.56, 3.12, 1.56 and 1.56 μg mL^−1^, respectively, along with compound **138a** active against *P. aeruginosa* (MIC 3.12 μg mL^−1^) as compared to the standard ampicillin. Compound **138a** was found active against Gram-negative strains (*S. aureus*, and *E. coli* with MIC 1.56 and 3.12 μg mL^−1^, respectively). The SAR of the synthesized compounds revealed molecules with halogen species could display antibacterial potential. Compound **138a** having chlorine species at para position of coumarin–benzimidazole framework found to have highest broad spectrum antimicrobial potential. 

**Scheme 30:**
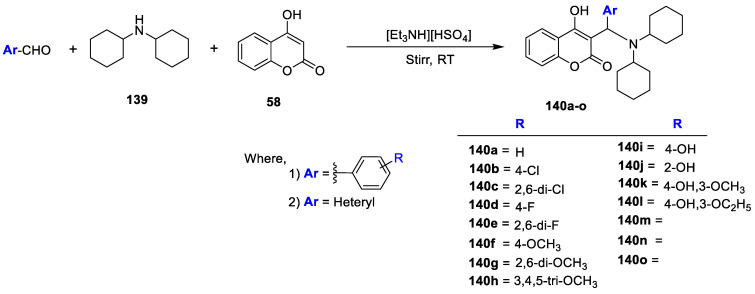
One—pot three—component synthesis routes for analogues **140a–o**. Adapted from Ref. [46]. under Creative Commons CC BY 4.0. Copyright 2017, MDPI.

Synthesis of novel 3-((dicyclohexylamino)-(substituted phenyl/heteryl)-methyl)-4-hydroxy-2H-chromen-2-onederivatives (**140a–o**) was carried out under solvent-free condition and there in vitro antimicrobial potential was evaluated by Shailee V. Tiwari et al. [46]. The title analogues were prepared via three component reactions of appropriate aldehydes, 4-hydroxy coumarin (**58**) and dicyclohexylamino (**139**) in the presences of [Et_3_NH] [HSO_4_] acting as a catalyst and a solvent (Scheme 30). The synthesized coumarin analogues were assessed for their antimicrobial potential against bacterial strains, namely, *E. coli*, *B. subtilis*, and *S. aureus*, and fungal strains *C. albicans*, *C. glabrata*, *F. oxysporum*, *A. fumigates*, *A. flavus*, *A. niger*, and *C. neoformans* via standard agar method using ampicillin and miconazole as reference drugs. Out of the test compounds, analogues **140e** having 2,4-difluoro group on the phenyl ring was found to be most active against *E. coli* (MIC 48 µg/mL), *B. subtilis* (MIC 50 µg/mL) and *S. aureus* (MIC 52 µg/mL). Furthermore, compound **140c** with 2,6-dichloro group on the phenyl ring has displayed excellent potency against *E. coli*, *B. subtilis* and *S. aureus* with MIC values 50, 48 and 50 µg/mL, respectively. The archived results revealed that analogues bearing electron-withdrawing substitutions such as **140e** (2,4-difluro), **140d** (4-fluro), **140c** (2,6-dichloro) and **140b** with 4-chloro on the phenyl ring with coumarin, dicyclohexylamine, and β-aminocarbonyl framework build up the antibacterial potential. Similarly, the obtained hybrids **140b**, **140c**, **140d** and **140e** with electron-withdrawing groups displayed significant in vitro antifungal potential against *A. fumigates*, *A. flavus* and *A. niger*. Interestingly, compound **140l** having 4-hydroxy-3-ethoxy on the phenyl ring has proven to be the most active antifungal agent from the series against *C. albicans*, *C. glabrata*, *Fusarium oxysporum*, *A. fumigates*, *A. flavus*, *A. niger*, and *C. neoformans* with MIC values 25, 28, 28, 36, 15, 12, and 12 µg/mL, respectively, while analogue **140e** seemed to be equally active against *A. fumigates*, *A. flavus*, *A. niger*, and *C. neoformans* when compared with the standard miconazole. 

**Scheme 31:**
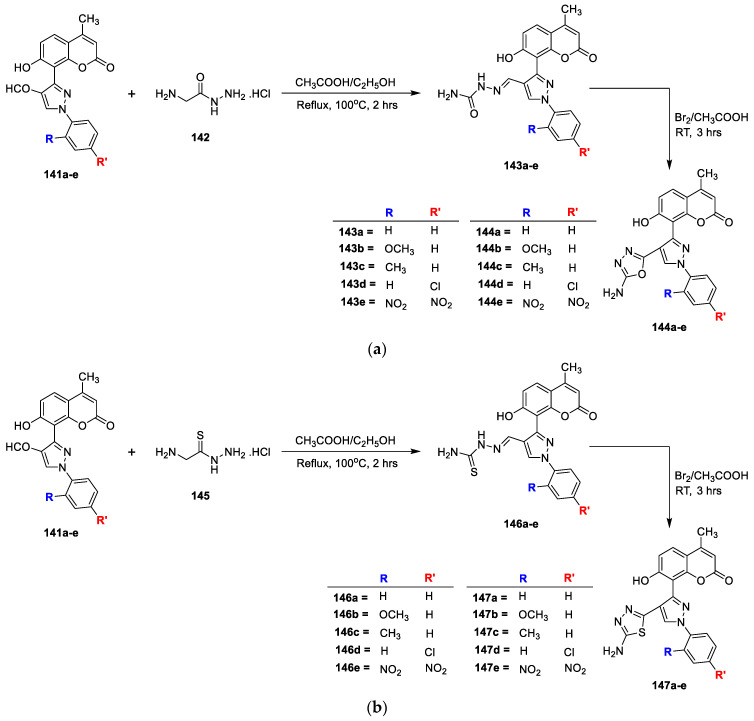
(**a**) Synthesis of coumarin–appended pyrazolyl-1,3,4-oxadiazoles **143a–e** and **144a–e**. (**b**) Route for the synthesis of coumarin–appended pyrazolyl-1,3,4-thiadiazoles analogues **146a–e** and **147a–e**. Reproduced and adapted with permission from Ref. [47]. Copyright 2017, Springer Nature.

Renuka et al. [47] reported antimicrobial and antioxidant activity of coumarin appended pyrazolyl-1,3,4-oxadiazoles and pyrazolyl-1,3,4-thiadiazoles. Initially, 1-aryl-3-(7-hydroxy-4-methyl-2- oxo-2H-chromen-8-yl)-1H-pyrazole-4-carboxaldehydes (**141a–e**) was treated with semicarbazide hydrochloride (**142**) in the presence of sodium acetate in ethanol under reflux to furnish the corresponding semicarbazones (**143a–e**), which is via oxidative cyclization using bromine in acetic acid gives 1,3,4-oxadiazoles (**144a–e**) (Scheme 31a). In the similar manner, precursors **141a–e** when reacted with thiosemicarbazide hydrochloride (**145**) in the presence of sodium acetate gives thiosemicarbazones (**146a–e**), which was converted into 1,3,4-thiaoxadiazoles (**147a–e**) on reaction with **146a–e** via oxidative cyclization using bromine in acetic acid (Scheme 31b). Among the series, chloro-substituted thiosemicarbazone was found to be excellent against all the tested pathogens, while methyl substituted thiosemicarbazone displayed good potential against *E. coli*. 

**Scheme 32:**
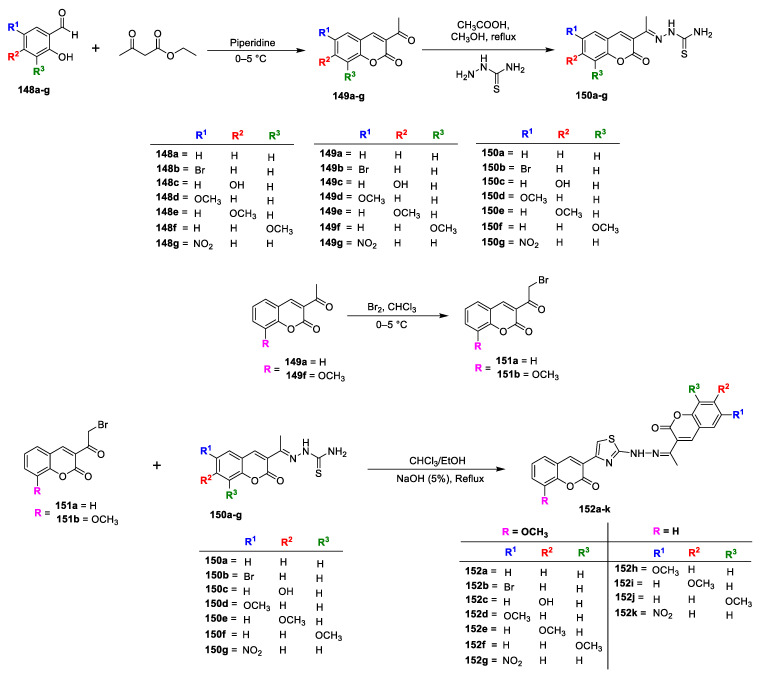
Synthesis of coumarin thiosemicarbazones **150a–g**, 3-bromocoumarin **151a**, **b**, and **152a–k**. Reproduced and adapted with permission from Ref. [48]. Copyright 2017, Springer Nature.

Samina Khan Yusufzai and co-workers [48] investigated in vitro antibacterial and antitubercular potential of some new hydrazinyl thiazolyl coumarin hybrids. Various 3-acetyl coumarins (**149a–g**) were prepared by the condensation of selective salicyladehydes (**148a–g**) with ethylacetoacetate in catalytic amount of piperidine, which on treatment with thiosemicarbazide furnishes coumarin thiosemicarbazone analogues (**150a–g**). The second precursor 3-(2-bromoacetyl)-2H-chromen-2-one (**151a,b**) was produced via bromination of 3-acetyl coumarins (**149a** and **f**). The title analogues **152a–k** were synthesized via Hantzsch cyclization of coumarin thiosemicarbazones (**150a–g**) and α-bromo-3-acetyl coumarins (**151a,b**) by cyclocondensation in CHCl_3_/EtOH followed by treatment with NH_4_OH (Scheme 32). In vitro antibacterial evaluation was carried out against *S. pneumoniae*, *S. aureus* (Gram-positive) and *E. coli*, *E. aerogenes*, *S. typhi* (Gram-negative) bacterial strains by employing colorimetric microdilution method. Among the newly furnished hybrids, Compound **152c** has displayed highest antibacterial potential against *S. typhi*, *S. pneumoniae*, and *S. aureus* with MIC values in the range of 31.25–62.5 μg/mL and are comparable to the standard kanamycin. Along with that compounds **152i**, **152j**, and **152k** has also exhibited good to moderate potential against all the tested pathogens with MIC in the range of 62.5–125 μg/mL. The SAR analysis has revealed that introduction of hydroxyl and halogens especially bromine on coumarin ring (**152c** and **152b**) could improve the antibacterial potential against all the pathogens compared to other substitutions. It was concluded that lipophilicity or hydrophobicity might be concerned with their mechanism of action. Halogens are highly reactive species due to their electronegativity thus, sufficient halogens (Cl, Br, and I) can be fatal to microorganisms. 

**Scheme 33:**
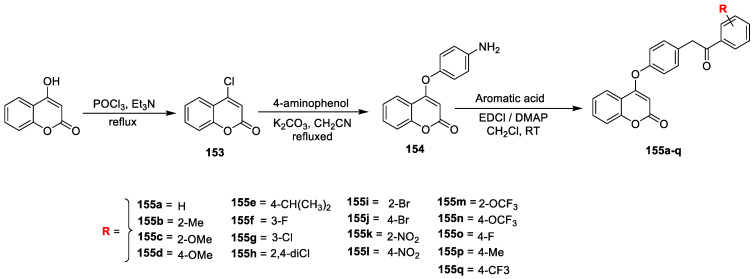
Synthetic route of the title compounds **155a–q**. Reproduced and adapted with permission from Ref. [49]. Copyright 2017, Springer Nature.

Mei-Hang Chen et al. [49] synthesized and screened several novel coumarin substituted amide derivatives (Scheme 33) as potential antibacterial agents against *Xanthomonas oryzaepv. Oryzae* (Xoo) and *Xanthomonas citri* subsp. *Citri* (Xcc). 4-hydroxycoumarin (**58**) was refluxed with POCl_3_ using Et_3_N reagent to yield 4-chlorocoumarin (**153**) via chlorination. Esterification of **153** with 4-aminoophenol in the presence of K_2_CO_3_ in CH_3_CN afforded 4-(4-aminophenoxy) coumarin (**154**). Finally, the compound **154** was treated with aromatic acid and 4-dimethylaminopyridin via condensation in CH_2_Cl_2_ gives amide derivatives containing coumarin (**155a–q**). The tested compounds **155f**, **155h**, **155k**, **155l**, **155m**, **155n**, **155o**, and **155q** found excellent antibacterial potency against *Xanthomonas oryzaepv. oryzae* at 200 μg mL^−1^ with percent zone of inhibition 92.7, 90.5, 97.3, 98.6, 94.1, 95.2, 92.4, and 96.0%, respectively, and were compared to the activity profile of the standard bactericide thiodiazole−copper (63.1%). Meanwhile, the same analogues displayed potential against Xoo at 100 μg mL^−1^ with percent inhibition of 55.1, 52.3, 55.7, 57.8, 52.1, 55.0, 52.6, and 56.3%, respectively, which is more than that of thiodiazole−copper (30.2%). Likewise, analogues **155f**, **155h**, **155k**, **155l**, **155m**, **155n**, **155o**, and **155q** has displayed acceptable antibacterial action against Xcc, with the inhibition rates of 93.8, 93.7, 98.1, 99.2, 96.1, 97.3, 93.2, and 97.0%, respectively, which is closer to the standard thiodiazole−copper (86.2%) at 200 μg mL^−1^. Based on the preliminary evaluation, the obtained EC_50_ value indicated that compounds **155f**, **155g**, **155h**, **155i**, **155j**, **155k**, **155l**, **155m**, **155n**, **155o**, and **155q** could display outstanding potency against Xoo (EC_50_: 135.8, 156.0, 145.7, 178.5, 160.2, 103.6, 96.4, 126.8, 119.0, 127.3, and 117.5 in all μg mL^−1^ respectively), while the same analogues exhibited significant potency against Xcc with EC_50_ of 115.0, 138.1, 101.2, 142.4, 136.8, 79.4, 73.2, 119.0, 112.4, 107.4, and 83, respectively, which is better than that of thiodiazole−copper (138.3 μg mL^−1^). The SAR study revealed that the electron-withdrawing groups F, Cl, Br, NO_2_, CF_3_, and OCF_3_ could display excellent potency against both tested pathogens (Xoo and Xcc) when compared to the electron-donating substitutions (Me, OMe) at the R position. From the above evidence, most of the amide bearing compounds with coumarin have shown improved potency against Xoo and Xcc than that of imine with the same coumarin moiety. 

**Scheme 34:**
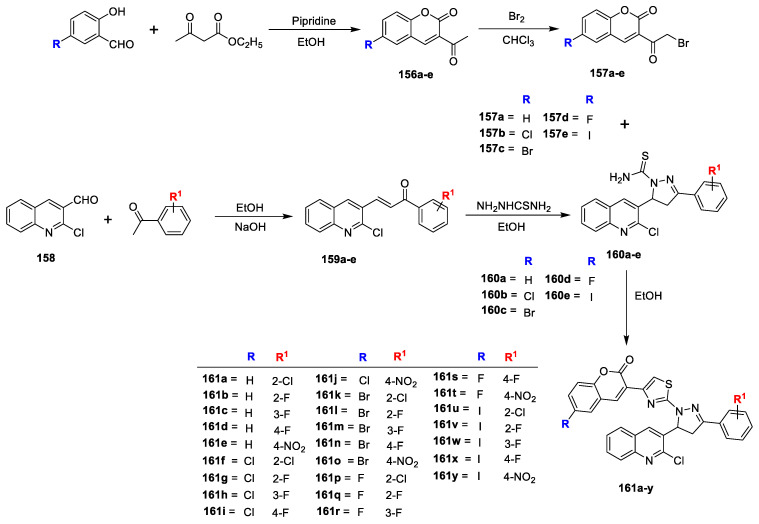
Systematic representation for synthesis of derivatives **161a–y**. Reproduced and adapted with permission from Ref. [50]. Copyright 2017, Springer Nature.

Mohd. Imran Ansari et al. [50] has reported a series of novel 3-(2-(5-(2-chloroquinolin-3-yl)-3-substituted phenyl-4,5-dihydro-1H-pyrazol-1-yl)-thiazol4-yl)-6-H/halo-2H chromen-2-ones and assessed for their in vitro antimicrobial activity. Compounds **156a–e** were prepared by reacting suitable substituted hydroxy benzaldehyde with ethylacetoacetate in catalytic amount of piperidine, which is via bromination to get converted into key intermediate 3-(2-bromoacetyl)-6-H/halo-2H-chromen-2-ones (**157a–e**). Meanwhile, 2-chloroquinoline-3-carbaldehyde on treatment with substituted acetophenone in ethanol obtained **159a–e**, which on treatment with hydrazine carbothioamide under reflux yields 5-(2-chloroquinolin-3-yl)-3-substituted phenyl-4,5-dihydro-1Hpyrazole-1-carbothiamide (**160a–e**). Finally, the title analogues **161a–y** were prepared via condensation of **157a–e** and **160a–e** in ethanol (Scheme 34). The prepared compounds screened for their in vitro antimicrobial action against *S. aureus*, *E. faecalis*, *Staphylococcus epidermidis*, *B. subtilis*, and *B. cereus* (Gram-positive), *E. coli*, *P. aeruginosa*, *K. pneumoniae*, *Bordetella bronchiseptica*, and *P. vulgaris* (Gram-negative) bacterial strain and five fungal strains, namely, *C. albicans*, *A. niger*, *A. flavus*, *Monascus purpureous*, and *Penicillium citrinum*, by using the serial plate dilution method. Some analogues **161q**, **161r**, and **161s** having fluoro-substituted coumarin on the phenyl ring has proven potential against tested bacteria as compared to the corresponding H/chloro/iodo/bromo-substituted compounds. Moreover, compounds **161q** and **161r** were found to possess excellent antifungal potential than that of the standard ofloxacin, and ketoconazole. 

**Scheme 35:**
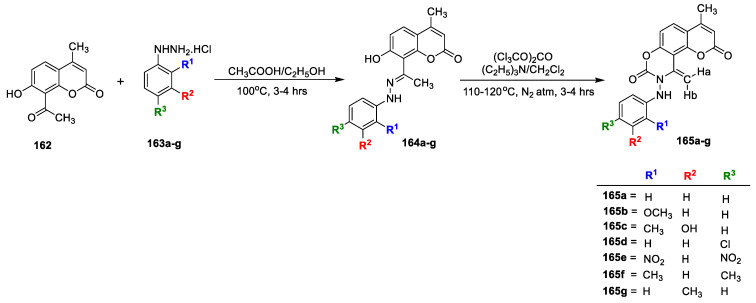
Scheme of the synthesis of oxazines **165a–g**. Reproduced and adapted with permission from Ref. [51]. Copyright 2017, Springer Nature.

In another study Renuka Nagamallu et al. [51] developed a series of coumarin appended 1,3-oxazines for their antimicrobial and antioxidant potential. The outline for the synthesis of title compounds is described in (Scheme 35). The corresponding hydrazones (**164a–g**) were synthesized via condensation of **162** with substituted phenylhydrazines (**163a–g**) in catalytic amount of AcOH under reflux. The title coumarins appended 1,3-benzoxazine analogues (**165a–g**) were prepared by treatment of corresponding hydrazones with triphosgene in the presence of Net_3_ and CH_2_Cl_2_ via cyclization. The obtained oxazine derivatives **165a–g** were evaluated for in vitro antimicrobial potential against *S. aureus*, *S. pyogenes*, *E. coli*, and *P. aeruginosa* bacterial strains and *C. neoformans*, *A. niger*, *A. flavus*, and *C. albicans* fungal strains. Compound **165b** having methoxy group exhibited potent activity against all the tested bacterial strains. After that compounds **165d** (chloro) and **165f** (dimethyl) have displayed greater extent of inhibition against *Streptococcus pyogenes* and *E. coli*. Compounds **165c** and **165g** with CH_3_ group have shown significant inhibitory potential against *S. pyogenes* and *E. coli*, whereas compounds **165b** (methoxy) and **165d** (chloro) had proved inhibitory potential against the tested fungi by inhibiting spore germination, while analogues **165c** and **165f** with a methyl group displayed good-to-moderate activity against *A. niger* and *A. flavus*. 

**Scheme 36:**
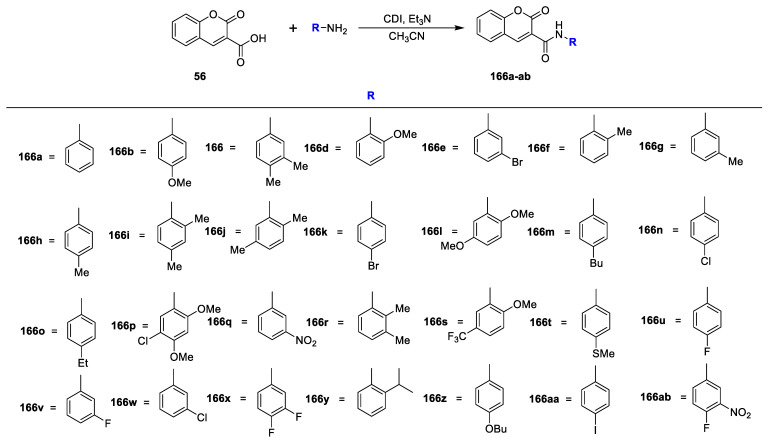
Development of coumarin-3-carboxamide derivatives **166a–ab**. Adapted from Ref. [52].

Uzma Salar et al. [52] synthesized and examined a series of coumarin-3-carboxamide derivatives for antimicrobial activity. The title analogues, coumarin-3-carboxamide (**166a–ab**) were synthesized form coumarin-3-carboxylic acid (**56**) with different substituted anilines in the presence of 1,1′-carbonyldiimidazole as a coupling agent (Scheme 36). In vitro antimicrobial activity was assessed against *B. subtilis*, *C. xerosis*, *S. aureus*, *S. faecalis*, and *MRSA* (Gram-positive) and *K. pneumoniae*, *P. aeruginosa*, *E. aerogene*, *and S. dysenteria* (Gram-negative) bacterial strains along with *A. niger*, *A. terrus*, *C. tropicalis*, *C. sacromyces*, *C. neoformen*, *C. albicans*, *Rhizopus*, *Microsporumcanis*, and *Absidia* fungal strains by using disc diffusion method. Compound **166ab** has exhibited highest antibacterial potential against *B. subtilis*, *C. xerosis*, *S. aureus*, *S. faecalis*, *MRSA*, *K. pneumoniae*, *P. aeruginosa*, *E. aerogene*, and *S. dysenteria*, along with **166b** and **166o**, while none of the derivatives were found active against the tested fungal strains. 

**Scheme 37:**
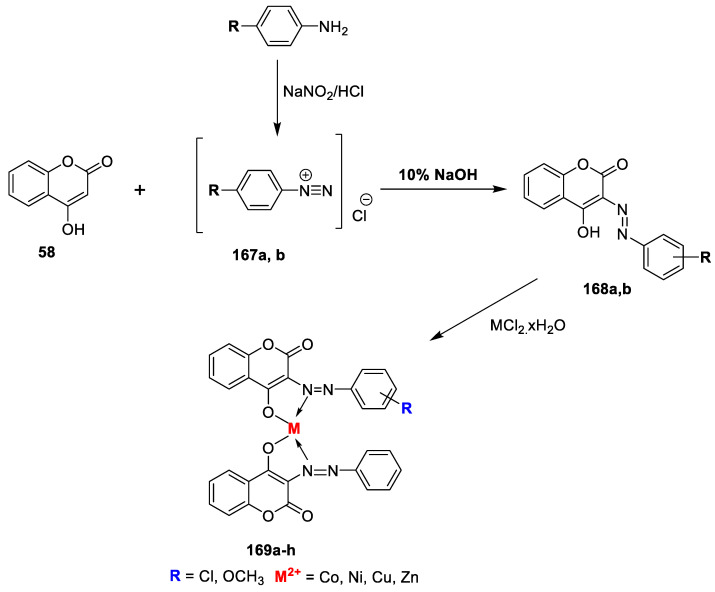
Coumarin-based transitional metal complexes **169a–h**. Adapted from Ref. [53]. under Creative Commons CC BY-NC-ND 4.0. Copyright 2016, Elsevier.

Jyotirmaya Sahoo et al. [53] has produced new transitional metal complexes derived form 3-aryl-azo-4-hydroxy coumarin analogues for their antimicrobial action. The metal complexes **169a–h** were yielded via condensation of different hydro alcoholic solutions of metal chlorides with 3-(4-chloro phenyl/4-methoxy phenyl)-azo-4-hydroxy coumarin derivatives (**168a,b**) (Scheme 37). The prepared complexes were assessed for antimicrobial potential against *E. coli*, *K. pneumonia*, *S. aureus*, *C. albicans* and *C. neoformans* and compared the derived results with standard ampicillin and fluconazole. Among all the synthesized complexes, only **169a** and **169e** exhibited better antimicrobial potential against *E. coli*, *K. pneumonia*, *S. aureus*, *C. albicans* and *C. neoformans* in comparison to the standard drugs (*p* < 0.05). 

**Scheme 38:**
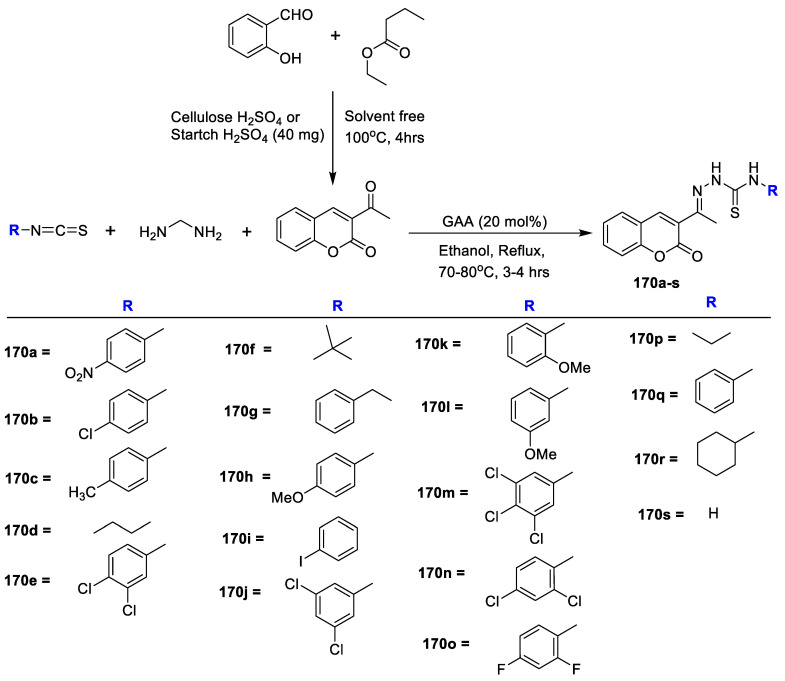
Novel coumarin-thiosemicarbazone derivatives **170a–s**. Adapted from Ref. [54]. under Creative Commons CC BY-NC-ND 4.0. Copyright 2016, Elsevier.

A series of 2-(1-(2-oxo-2Hchromen-3-yl) ethylidene) hydrazinecarbothioamide derivatives were contributed by R H. Vekariya et al. [54] in order to assess their antimicrobial activity (Scheme 38). Salicylaldehyde on reaction with ethyl acetoacetate in the presence of starch sulfuric acid or cellulose sulfuric acid gives 3-acetyl coumarin under solvent free conditions. The obtained 3-acetyl coumarin was treated with isothiocyanatobenzene and hydrazine hydrate in the presence GAA under reflux to furnish title coumarin-clubbed thiosemicarbazone derivatives (**170a–s**). The prepared hybrids were screened for their in vitro antimicrobial activity against bacterial strains (*S. aureus*, *S. pyogenes*, *E. coli*, *P. aeruginosa*) and fungal strains (*A. clavatus*, *C. albicans*, and *A. niger*) by employing agar dilution method using ampicillin, ciprofloxacin, chloramphenicol, nystatin and griseofulvin as standard control drugs. Biological studies have revealed that compound **170o** with electron deactivating fluoro group on phenyl (2 and 4 positions) has excellent potential against *S. aureus* with MIC 50 lg/mL. Analogues **170b**, **170e**, **170j**, and **170m** bearing chloro group at phenyl ring were found excellent inhibitors next to **170o** against *S. aureus* with MIC 125 lg/mL. Moreover, candidates **170b**, **170e**, **170h**, **170j**, **170m**, **170n**, and **170s** were found as potent inhibitors against *E. coli* at MIC of 62.5 lg/mL. On the other hand, compound **170o** bearing fluoro substitution at phenyl was found active against fungi *C. albicans* with MIC value 100 lg/mL which is equal to the standard nystatin and greater than that of griseofulvin, while analogues **170b**, **170e**, **170j**, **170m**, and **170n** having a chloro group at phenyl displayed potency against *C. albicans* with MIC 250 lg/mL. Moreover, analogue **170i** with an iodo group at the para position of the phenyl ring had proven better activity against *C. albicans* (MIC 250 lg/mL) compared to the standard drugs. 

**Scheme 39:**
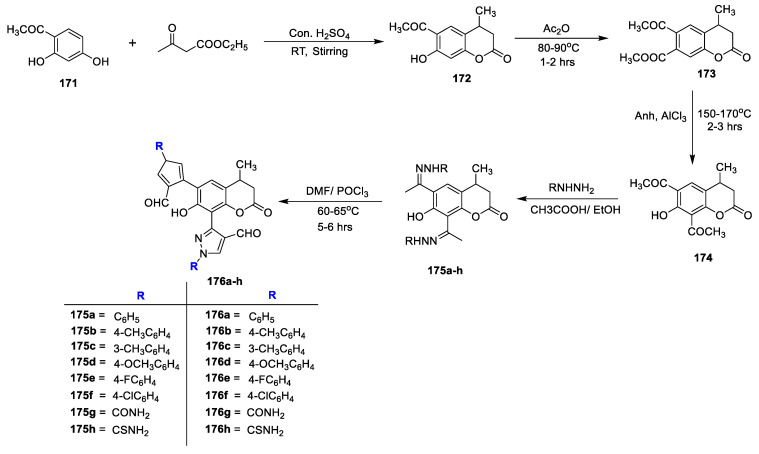
Design of coumarin—appended bis—(formyl pyrazoles). Reproduced and adapted with permission from Ref. [55]. Copyright 2017, Elsevier.

In continuing efforts to find potent antimicrobial candidates R. Nagamallu and co-workers [55] reported novel coumarin appended bis (formyl pyrazole) derivatives for their antimicrobial and antioxidant potential. The outline of synthesis is displayed in (Scheme 39). Initially, 6-acetyl-7-hydroxy-4-methyl-2H-chromen-2-one (**172**) was furnished via Pechmann reaction of 1-(2, 4-dihydroxyphenyl) ethanone (**171**), and ethyl acetoacetate. The obtained **172** was treated with acetic anhydride to yield 6-acetyl-4-methyl-2-oxo2H-chromen-7-yl acetate (**173**) which was subjected to Fries rearrangement using AlCl_3_ to give 1,1′-(7-hydroxy-4-methyl-2-oxo-2H-chromene-6,8-diyl) diethanone (**174**). The prepared **174** was reacted with substituted phenylhydrazines, semicarbazine, and thiosemicarbazine in ethanol and catalytic amount of glacial acetic acid (GAA) was refluxed to yield corresponding bis-hydrazones **175a–h** followed by conversion to label coumarin appended bis (formyl pyrazole) (**176a–h**) under Vilsmeier-Haack conditions. The biological assays were performed against *E. coli*, *P. aeruginosa* and *S. aureus* bacterial strains, as well as *A. niger*, *A. flavus*, and *C. albicans* via the broth dilution method using ciprofloxacin and fluconazole as the standard drugs. Most of the analogues from the series were found moderately active against tested bacterial strains with MIC values ranging from 6.25–75 µg/mL. Analogues **175g** and **175h** with CONH_2_ and CSNH_2_ exhibited excellent antibacterial activity against selected bacteria and compounds **176a** and **176h** with same group at pyrazole ring were found potent as compared to the standard. Compounds **175d** and **175b** bearings methoxy and methyl group have displayed good to moderate activity. All the test compounds were further tested for antifungal potential and compounds **175g** and **175h** with CONH_2_ and CSNH_2_ revealed excellent potential, while **176g** with CONH_2_ has shown potency similar to standards and the hybrid **176h** having CSNH_2_ was found potent against *A. niger*. 

**Scheme 40:**
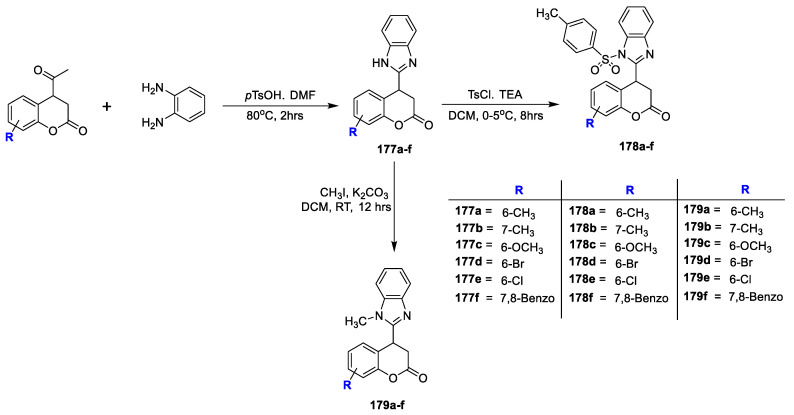
Synthetic route for the coumarin—benzimidazole hybrids. Reproduced and adapted with permission from Ref. [56]. Copyright 2016, John Wiley and Sons.

Megharaja Holiyachi et al. [56] in another study demonstrated the antibacterial and anticancer activity of coumarin benzimidazole hybrids. The basic skeleton of coumarin-benzimidazole (**177a–f**) were obtained by rection of 4-formylcoumarins with ortho-phenylenediamine in the presence of p-toluene sulphonic acid in DMF under reflux. Further the resulting intermediate was converted to coumarin-benzimidazole sulphonamide analogues (**178a–f**) by treatment of *p*-toluenesulfonyl chloride with **177a–f** via N-sulphonation. On the other hand, obtained **177a–f** on reaction with methyl iodide in the presence of anhydrous potassium carbonate yield **179a–f** (Scheme 40). All the furnished derivatives were screened against *Bacillus flexus* (Gram-positive) and *Pseudomonas* spp. (Gram-negative) bacteria. Compounds substituted with **177c** (OCH_3_), **177d** (Br) and **177e** (Cl) at C-6 of coumarin moiety were found to be highly active against both the bacterial strains as compared to the standard ciprofloxacin. Among the series **178a–f** and **179a–f**, analogues **178c**, **178d** and **178e** have displayed excellent potency against both the tested pathogens, whereas N-methylated analogues were found less active except **179e** having chloro substitution. For antifungal potency, compound **117e** with chloro substitution at C-6 of coumarin was found active against *Scopulariopsis* spp., while different substitutions at C6 of the coumarin moiety in compounds **178c** (OCH_3_), **178d** (Br), and **178e** (Cl) had proven promising results against both *Scopulariopsis* spp. and *Aspergillus tereus*. Thus, the obtained results revealed that presence of larger group such as OCH_3_, Cl and Br at C-6 of coumarin framework are responsible to show potent antibacterial and antifungal potential, whereas, for series **177a–f**, N-sulphonation did not reveal any improvement in the potency; however, N-methylation reduced the potential. 

**Scheme 41:**
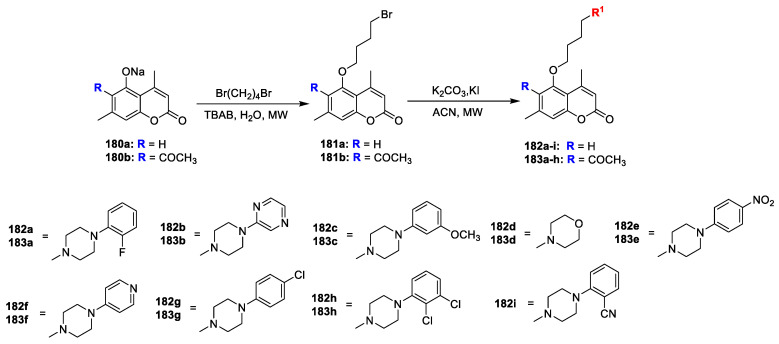
Route for the development of 5-[4-(4-aryl-1-piperazinyl) butoxy] coumarins. Reproduced and adapted with permission from Ref. [57]. Copyright 2016, Springer Nature.

Kinga Ostrowska et al. [57] investigated antitumor and antimicrobial potential of 5-[4-(4-aryl-1- piperazinyl)butoxy]coumarins against *M. luteus*, *B. subtilis*, *B. cereus*, *S. aureus*, and *E. hirae* (Gram-positive) and *E. coli* and *P. aeruginosa* (Gram-negative) bacterial strains, as well as *C. albicans* and *C. parapsilosis* fungal strains via the modified cylinder-plate method. The intermediate 1-(4,7-dimethylcoumarin-5-yloxy)-4-bromobutane (**181**) and 1-(5-acetyl-4,7-dimethylcoumarin-5-yloxy)-4-bromobutane (**181b**) were prepared by reaction of 5-hydroxy-4,7-dimethylcoumarin (**180a**) or 6-acetyl-5-hydroxy-4,7-dimethylcoumarin (**180b**) with sodium hydroxide in toluene by using 1,4-dibromobutane, tetrabutylammonium bromide alkylating agents via microwave. The obtained bromoalkyls (**181a** and **181b**) with N-substituted piperazine derivatives in the presence of potassium carbonate in acetonitrile yield title analogues (**182a–I** and **183a–h**, respectively) (Scheme 41). The two analogues **182f** and **183f** displayed good antibacterial potency. It was observed that 4,7 dimethyl-5-[4-[4-(1-(4-pyridyl)) piperazin-1-yl] butoxy] coumarin (**182f**) was active against *M. luteus* with MIC 15 µg/cm^3^. Another 5-acetyl-4,7- dimethyl-5-[4-[4-(1-(4-pyridyl)) piperazin-1-yl] butoxy] coumarin (**183f**) with acetyl substitution was found less active, while other compounds were found inactive. Overall, all the analogues bearing methoxy substituents on aromatic ring or 4-pyridyl substituent were found active against tested organisms. Similarly, Compounds **182f** and **183f** were found active against tested fungi. 

**Scheme 42:**
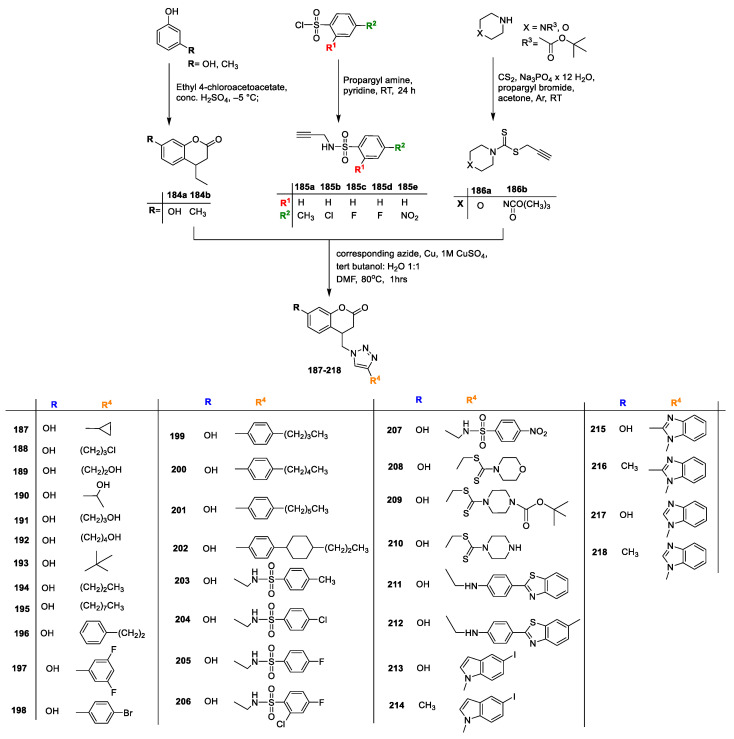
Synthesis of the 1,2,3-triazole-coumarin hybrids. Reproduced and adapted with permission from Ref. [58]. Copyright 2016, Elsevier.

Tatjana Gazivoda Kraljević and co-workers [58] reported anticancer and antibacterial activity of some 4-substituted 1,2,3-triazole–coumarin hybrids. In vitro antibacterial evaluation was done against bacterial strains, namely, *S. aureus*, *E. faecalis*, and *E. faecium* (Gram-positive) and *P. aeurigonsa*, *E. coli*, *A. baumannii*, and *K. pneumoniae* (Gram-negative), and the obtained results were compared to the standard ceftazidime and ciprofloxacin. The title compounds 1,2,3-triazole–coumarin hybrids (**187–218**) were furnished via Huisgen 1,3-dipolar cycloaddition reaction of a terminal alkyne with an azide using copper(I) as the catalyst (Scheme 42). The synthesized analogues failed to display considerable antibacterial potential against both tested bacterial species, with the exclusion of some selective activity against *Enterococcus* species. The obtained results have shown that coumarin–1,2,3-triazole analogues bearing substituted phenyl rings (**196–202**), hybrids having p-alkyl phenyl (**199–201**), and (p-alkylcyclohexyl) phenyl (**202**) at 4-substituted 1,2,3-triazole nucleus exhibited promising potency against *Enterococcus faecalis* with MICs values ranging from 8–64 µg/mL. Among the 4-(benzene sulphonamide) methyl-1,2,3-triazole–coumarin series, compounds having p-methyl (**203**) and p-fluorobenzene sulphonamide subunits (**205** and **206**) have proven their potential against *E. faecalis* with MICs 64 and 32 µg/mL. In addition to that, compounds **208** (morpholine) and **210** (piperazine) displayed significant potential with MICs 32 µg/mL and 16 µg/mL, respectively. 

**Scheme 43:**
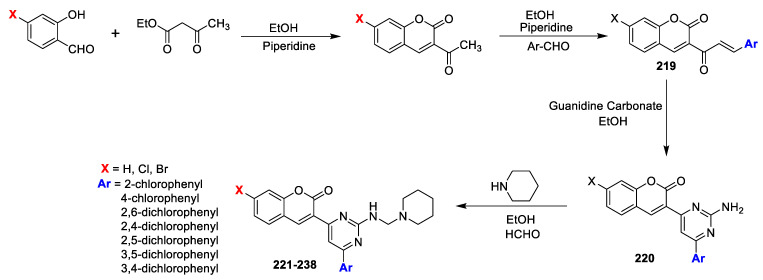
Designing of coumarin derivatives **221–238**. Adapted from Ref. [59].

A series of 2-piperidinomethylamino-4-(7-H/substituted coumarin-3-yl)-6- chloro substituted phenyl pyrimidines was prepared and screened for antimicrobial potential by Mohd Imran et al. [59]. Title analogues **121–238** were furnished by reactions of 2-amino-4-(7-H/substitutedcoumarin-3-yl)-6-(chlorosubstitutedphenyl) pyrimidines (**220**) with piperidine and formaldehyde (Scheme 43). Among the synthesized series, compound **226** was found to be highly potent against *S. aureus*, *E. faecalis*, *S. epidermidis*, *B. subtilis*, and *B. cereus* with MIC 50 μg/mL (*p* < 0.05 or less). Compound **226** has exhibited significant activity against tested Gram-negative bacterial strains (*E. coli*, *P. aeruginosa*, *K. pneumonia*, *B. bronchiseptica*, and *P. vulgaris*) with MIC value 25 μg/mL (*p* < 0.05 or less). Moreover, compounds **226** and **237** have proven excellent inhibitors against tested fungi (*C. albicans*, *A. niger*, *A. flavus*, *M. purpureous*, and *P. citrinum*) with MIC 25 μg/mL (*p* < 0.0001) and 25 μg/mL (*p* < 0.0001), respectively. The derived SAR concludes that the presence of bromo substitution at 7-position of coumarin ring along with 4-chlorophenyl at 6-position of the pyrimidine ring has played a critical role for inhibition of tested bacterial and fungal strains.

**Scheme 44:**
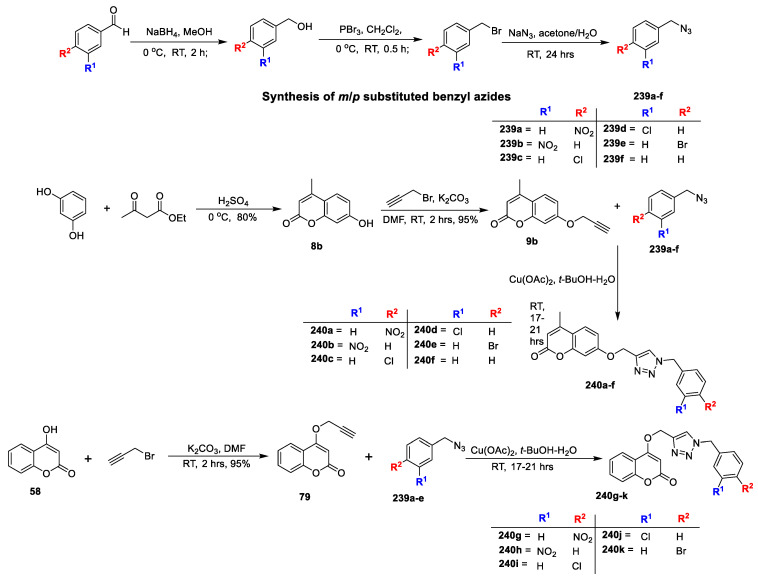
Synthesis of 1,4-disubstituted-1,2,3-triazole-based coumarin derivatives **240a–k**. Reproduced and adapted with permission from Ref. [60]. Copyright 2016, Springer Nature.

M.H. Shaikh et al. [60] developed a series of coumarin incorporated triazoles analogues and assessed for their antitubercular, antioxidant and in vitro antimicrobial activity. The title analogues were synthesized by procedure depicted in (Scheme 44). Initially the starting material required for synthesis of label analogues was benzyl azides and **239a–f** were furnished by reaction of corresponding benzaldehydes with sodium azide via NaBH_4_ reduction, bromination, and nucleophilic substitution reaction. The 7-hydroxy-4-methyl coumarin (**8b**) obtained via Pechmann condensation, reaction of **8b** and **58** with propargyl bromide in the presence of K_2_CO_3_ in DMF afforded 4-Methyl-7- (prop-2-yn-1-yloxy)-2H-chromen-2-one (**9b**), and 4-(Prop-2-yn-1-yloxy)-2H-chromen-2-one (**79**), respectively. In the final step, prepared benzyl azides (**239a–f**) and coumarin-based alkynes (**9b** and **79**) via 1,3-dipolar cycloaddition reaction in the presence of t-BuOH-H2O (1:1) afforded corresponding 1,4-disubstituted-1,2,3-triazolebased coumarin derivatives (**240a–f** and **240g–k**, respectively). The test compounds were subjected to in vitro antimicrobial evaluation against *S. aureus*, *M. luteus*, and *B. cereus* (Gram-positive) *E. coli*, *P. fluorescens*, and *F. devorans* (Gram-negative) bacterial strains, as well as *A. niger*, *P. chrysogenum*, and *C. lunata* fungal species via serial dilution method using kanamycin, ampicillin, chloramphenicol, miconazole, fluconazole, and amphotericin B as the standard drugs. Among the series, most the analogues were good inhibitors against all the tested bacterial and fungal strains. Among the series, compounds **240g** and **240d** revealed excellent antimicrobial potential against all the tested pathogens as compared to the standard analogues. 

**Scheme 45:**
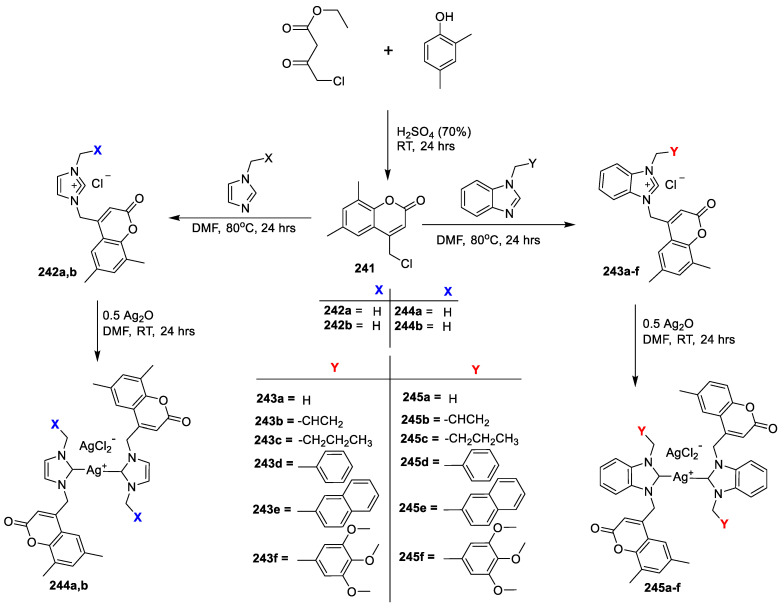
Development of imidazolium **242a**,**b** benzimidazolium **243a–f** chlorides and silver–NHC complexes **244a**,**b** and **245a–f**. Reproduced and adapted with permission from Ref. [61]. Copyright 2015, Elsevier.

Mert Olgun Karatas et al. [61] evaluated antimicrobial activity of novel silver (I) complexes with coumarin substituted N-heterocyclic carbene ligands. Novel eight coumarin substituted silver(I) N-heterocyclic carbene (NHC) complexes were produced via the interaction of the corresponding imidazolium or benzimidazolium chlorides and Ag_2_O in dichloromethane (Scheme 45). All the synthesized complexes were screened for their antimicrobial potency against *E. faecalis*, *S. aureus*, *E. coli*, and *P. aeruginosa* bacterial strains and *C. albicans* and *C. tropicalis* fungal stains. The obtained results demonstrated that about all the complexes had the potential to inhibit the growth of all the tested pathogens, while some of them displayed good action against different microorganism as compared to the standard analogues. From the tested complexes, the most lipophilic compound **245e** (bis [1-(4-methylene-6,8-dimethyl-2H-chromen-2-one)-3-(naphthalene-2-ylmethyl) enzimidazole-2-ylidene] silver(I) dichloroargentate) was found to be most active as compared to the standard next to **245d**. 

**Scheme 46:**
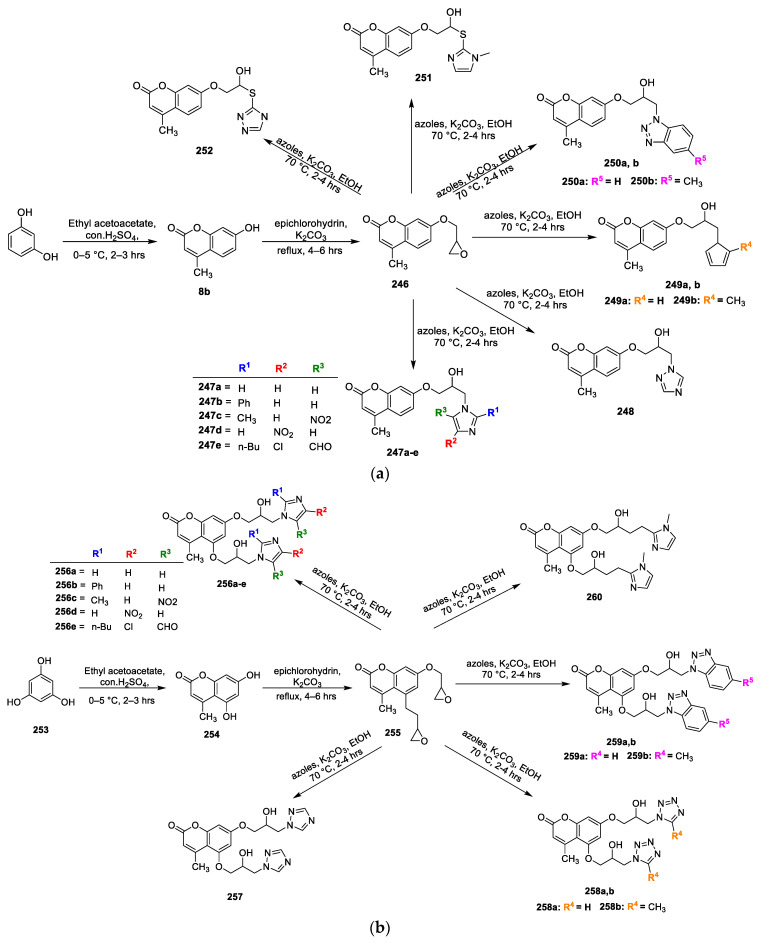
(**a**) Coumarin-clubbed mono-azolyl ethanols. (**b**) Coumarin-clubbed bis-azolyl ethanols. Reproduced and adapted with permission from Ref. [62]. Copyright 2016, Springer Nature.

Xin-Mei Peng and co-workers [62] reported antimicrobial activity of some new coumarin-derived azolyl ethanols including imidazolyl, triazolyl, tetrazolyl, benzotriazolyl, thiol-imidazolyl, and thiol-triazolyl. The synthetic strategies to prepare title compounds coumarin-derived azolyl ethanols is depicted in (Scheme 46a,b). Key intermediates **246** and **255** were synthesized via O-alkylation of 2-(chloromethyl) oxirane with hydroxyl coumarins (**8b** and **254**, respectively). The open ring compounds with suitable azoles using K_2_CO_3_ as base in ethanol gives corresponding mono-azolyl ethanols (**247–252**) and bis-azolyl ethanols (**256–260**). The resultant analogues evaluated for in vitro antimicrobial potential against Methicillin-resistant *S. aureus*, *B. subtilis*, *M. luteus* (Gram-positive) and *E. coli*, *P. aeruginosa*, and *S. dysenteriae* (Gram-negative) bacterial strains, as well as fungi (*C. albicans*, *B. yeast*, *C. utilis*, *C. mycoderma*, and *A. flavus*) via the two-fold serial dilution method. Antibacterial screening has revealed that some of the analogues could display good to moderate potential against the selected bacterial strains as compared to the standard chloramphenicol and norfloxacin. Among the series, compound **257** bearing bis-triazolyl ethanol has displayed low MCI value of 8 mg/mL against MRSA, which was equivalent or better than that of standard drug norfloxacin MIC 8 mg/mL and chloramphenicol MIC 16 mg/mL. Compound **257** also found promising to inhibit growth of all tested fungi as compared to standard fluconazole.

**Scheme 47:**
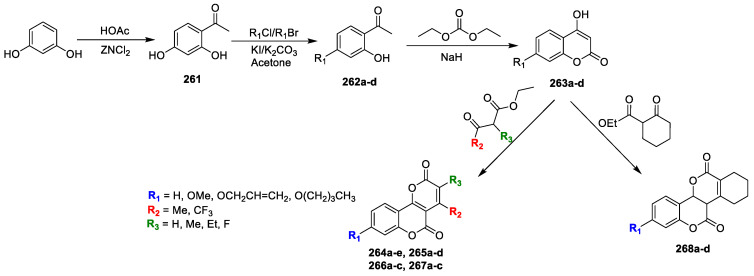
Synthetic route for target compounds **264a–268d**. Reproduced and adapted with permission from Ref. [63]. Copyright 2016, Elsevier.

R.R. Zhang et al. [63] performed microwave assisted synthesis of some coumarin fused pyrano [3,2-c] chromene-2,5-diones (Scheme 47) and the results of antifungal action against *Botrytis cinerea*, *Colletotrichum copsica*, *Alternaria solani*, *Gibberella zeae*, and *Rhizoctorzia solani* were compared to a marketed broad-spectrum fungicide azoxystrobin. The prepared 3-(trifluoro) methyl **364a–e**, 8-alkoxy **265a–d**, 8-allyloxy **266a–c**, pyrano[3,2-c] chromene-2,5-diones **267a–c** and 3,4-cyclohexane pyrano[3,2-c] chromene-2,5-diones **268a–d** evaluated for their fungicidal potential. Among the series, some of the analogues found to be potent inhibitors of *Botrytis cinerea* and *Collecterichum* capsica at conc < 50 ppm. Compounds **264d**, **265c**, and **266b** were found to be highly active against *Botrytis cinerea* with EC_50_ values 0.141, 0.082, and 0.091 µM, respectively, which is much better than the standard drug azoxystrobin. Additionally, compound **264d** (EC_50_ = 0.115 µM) also found to be an excellent inhibitor against *Collecterichum capsica* as compared to reference (EC_50_ = 0.222 µM). Therefore, from above biological results it can be concluded that **264d** is highly active and most promising compound for further studies.

**Scheme 48:**
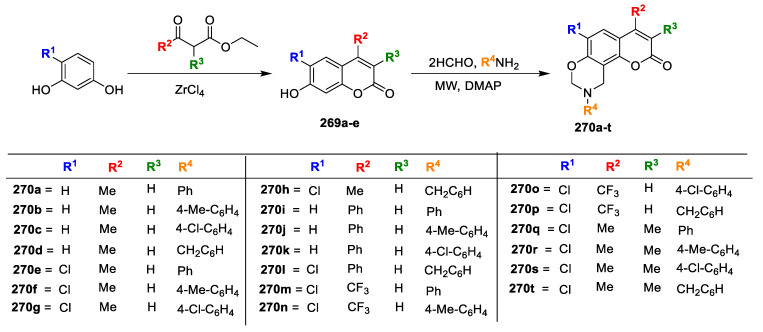
Synthetic route for **270a–t**. Reproduced and adapted with permission from Ref. [64]. Copyright 2016, Springer Nature.

In another study R.R. Zhang and co-workers [64] investigated antifungal properties of some coumarin[8,7-e] [1,3] oxazine derivatives. Initially coumarins (**269a–e**) were synthesized via Pechmann condensation using ZnCl_4_ as the catalyst, which, on reactions with different and catalytic amounts of 4-N, N, dimethylaminopyridine under microwave, afforded target analogues **270a–t** (Scheme 48). The antifungal potency of these analogues was evaluated at concentration 50 ppm using reference drug osthole. Experimental studies revealed that compounds **270e**, **270m**, and **270s** possess inhibitory potency against *Botrytis cinerea* with zone of inhibition 88.2, 98.7, and 78.6, respectively, and are comparable to the control analogues and osthole (86.8%), while compounds **270g** and **270m** showed growth in their inhibition against *Colletotrichum capsica* with 66.7 and 79.6%, respectively. However, analogues **270e**, **270m**, and **270o** revealed 73.3, 88.2, and 81.5% inhibition, respectively, against *Rhizoctonia solani*.

## 3. Coumarin-Based Natural Products as Antimicrobial Agents

Plant species is one of the rich sources of novel biologically active analogues [65]. Plant-based compounds have benefited traditional medicines in the management of diseases. Worldwide population of around 60% rely on use of medicinal plants as primary healthcare regimen. Nowadays various medicinal herbs are being evaluated for their pharmacological potential. Coumarins were originally derived from plants isolated by Vogel in 1820 from tonka bean *Dipteryx odorata* (Aubl.) Willd. Coumarins derivatives occurs in every part of the plant species from roots to flowers and fruits. These are widely distributed in several plant families including Leguminosae, Orchidaceue, Umbelliferae, Rutaceae, Apiaceae, Clusiace, Caprifoliaceae, Oleaceae, Guttiferae, Nyctaginaceae, and grasses [6,66]. The isolated coumarins have displayed broad spectrum pharmacological potential. In this section, we discuss research outcomes reported in the duration 2016–2021 including coumarins analogues isolated from plant sources having antimicrobial potential.

**Figure 10:**
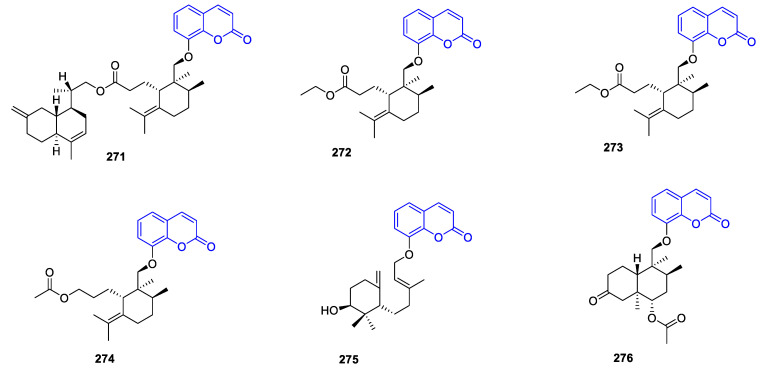
Isolated coumarins from *F. pseudalliacea* roots **271** sanandajin, **272** methyl galbanate, **273** ethyl galbanate, **274** fekrynol acetate, **275** farnesiferol B, and **276** kamonolol acetate.

Dara Dastan et al. [67] evaluated antibacterial potential of disesquiterpene and sesquiterpene coumarin analogues isolated from *Ferula pseudalliacea* roots. Antibacterial studies were performed using seven bacterial strains, namely, *S. aureus*, *Enterococcus faecium*, *Bacillus cereus*, *E. coli*, *Pseudomonas aeruginosa*, *K. pneumoniae*, and *Helicobacter pylori*, through the broth micro-dilution method, as per the standard CLSI protocol. Among the isolated analogues, sanandajin (**271**) and ethyl galbanate (**273**) were found active against *H. pylori* and *S. aureus* at a concentration of 64 μg/mL. Similarly, methyl galbanate (**272**) have exhibited significant potential against vancomycin resistant strain of *E. faecium* at a concentration of 64 μg/mL (Figure 10).

**Figure 11:**
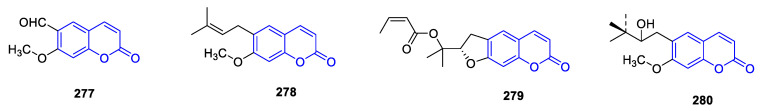
Chemical structures of the isolated coumarins from rhizomes of *Ferula gotrachycarpa* Boiss (**277–280**).

Tuğçe Dikpınar and co-workers [68] isolated four known coumarins, crenulatin (**277**), suberosin (**278**), marmesin seneociate (**279**), and Ulopterol (**280**) (Figure 11), from *Ferula gotrachycarpa* Boiss and screened them for antimicrobial activity against bacterial strains (*S. aureus*, *S. epidermidis*, *E. coli*, *K. pneumoniae*, *P. aeruginosa*, *P. mirabilis*, and *E. feacalis*) and fungal strains (*C. albicans*, *C. tropicalis*, and *C. parapsilosis*) by microdilution method as per CLSI guidelines (CLSI 2000, 2006). All the four isolated extract were found active against tested pathogens. Crenulatin (6-formyl-7-methoxycoumarin) (**277**), suberosin (7-methoxy-6-prenylcoumarin), and marmesin senecioate ((-)-prantschimgin) (**278**) demonstrated antifungal potential against *C. albicans* with MIC value of 625 mg/L, and antibacterial activity with MIC 1250 mg/L against *S. aureus* (MRSA). If compared to the generalized categorization of active/less active based on MIC [16] these identified compounds (MIC > 100 μg/mL) cannot be considered as potential antimicrobial agents.

**Figure 12:**
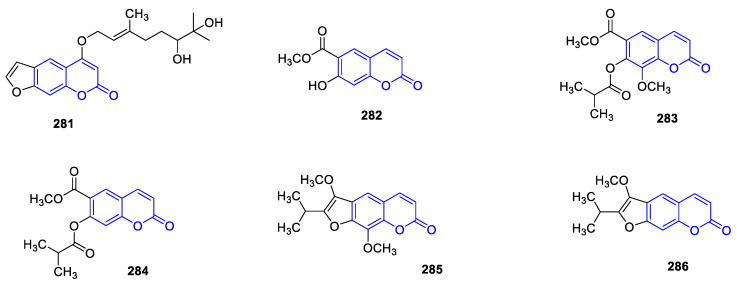
Coumarins moieties where isolated from *P. luxurians* 6′,7′-dihydroxybergamottin **281**, officinalin **282**, stenocarpin isobutyrate **283**, officinalin isobutyrate **284**, 8-methoxypeucedanin **285**, and peucedanin **286**.

Six coumarin derivatives, 6′,7′-dihydroxybergamottin (**281**), officinalin (**282**), stenocarpin isobutyrate (**283**), officinalin isobutyrate (**284**), 8-methoxypeucedanin (**285**), and peucedanin (**286**) were isolated from the fruits of *Peucedanum luxurians* tamamsch (Figure 12). Antimicrobial activities of extracts and isolated coumarins was assessed against *S. aureus* and *S. epidermidis* (Gram-positive) and *P. aeruginosa*, *E. coli*, *E. cloacae*, *and K. pneumoniae* (Gram-negative) using diffusion and dilution methods. All the isolated extracts have displayed extensive ranges concerning to inhibitory activity against tested microorganism with 6′,7′-dihydroxybergamottin (**281**) was found to be most active against all tested bacteria with zone of inhibition ranging between 16 and 17 mm with MIC values between 1.20 and 2.10 mg/mL. Similar potential was expressed by peucedanin (**286**) and officinalin isobutyrate (**284**) against tested bacterial stains with zone of inhibition ranging between 16 to 13 mm and MIC values between 1.40 and 4.80 mg/mL, whereas the other extracted coumarins revealed good to moderate potential with zone of inhibition between 12 to 14 mm with MIC values ranging from 3.90 to 5.75 mg/mL [69].

**Figure 13:**
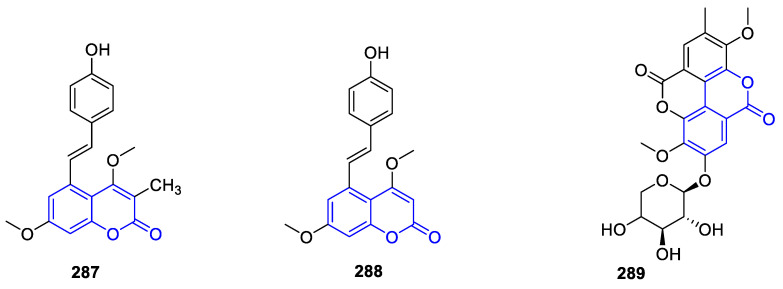
Chemical structures of coumarins isolated from the stem barks and leaves of *Monotes kerstingii Gilg* (**287–289**).

Nineteen secondary metabolites isolated from the stem, bark and leaves of *Monotes kerstingii* by Ghislain Wabo Fotso et al. [70]. Isolated extract was screened for in vitro antimicrobial potential against bacteria (*Bacillus subtilis*) and fungi (*Septoria tritici*) using erythromycin, epoconazole, and tabernafine as positive controls. Antibacterial results demonstrated that, compounds 1-{2-hydroxy-6-[(1E)-2-(4-hydroxyphenyl) ethenyl]-4,7-dimethoxy2H-1-benzopyran-2-one (**287**), 5-[(1E)-2-(4-hydroxy phenyl) ethenyl]-4,7- dimethoxy-3-methyl-2H-1-benzopyran-2-one (**288**), **and** 3,3′-di-O-methylellagic acid-4′-O-β-D-xylopyranoside (**289**) (Figure 13) exhibit significant potential against *B. subtilis* at concentration 100 μM with zone of inhibition 99, 79, 71, and 100%, respectively, when compared to the reference erythromycin.

**Figure 14:**
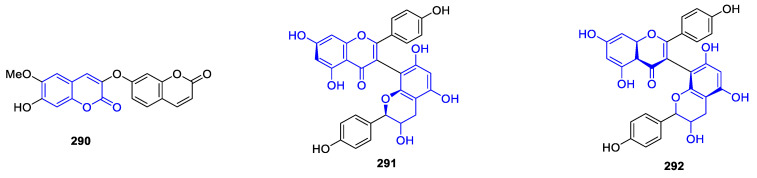
Chemical structures of coumarins isolated from *Gymnocarpos decandrus* Forssk roots (**290–292**).

The antimicrobial potential of daphnoretin (**290**), wikstrol A (**291**), and wikstrol B (**292**) isolated from *Gymnocarpos decandrus* Forssk roots (Figure 14) was screened against *Bacillus subtilis* and *Staphylococcus aureus* (Gram-positive) and *E. coli* and *Pseudomonas aeruginosa* (Gram-negative) bacterial strains, as well as the fungi *Candida albicans*. The test extracts were found to possess significant inhibitory potential against *Bacillus subtilis* [71]. Thus, the above collection suggests that coumarin analogues isolated from their natural origins form a significant yet new class of antimicrobial compounds. The modification in their structures could lead to more effective antimicrobial agents.

## 4. Patents

The coumarin hybrid and its derivatives have potential antimicrobial activity, and research is ongoing in the coumarin motif, signifying it is a privileged scaffold for the design and development of new potent antimicrobial agents. This section deals with some important patents granted to coumarin motifs for their antimicrobial potential (Table 1).

## 5. Conclusions

A wide range of biological activities have been reported for coumarin-based derivatives, and some coumarin-based hybrids have been used to treat various infections. In the last few decades, various coumarin analogues were developed and screened for Gram-negative, Gram-positive, and MDR microorganisms (in vivo and in vitro). In this context, the current developments in coumarin derivatives were covered. The structural characteristics related to the antimicrobial properties of synthetic coumarins were intensely explored for modifications/substitutions at the C-3, C-4, C-6, and C-7 positions. A review of the structural characteristics highlighted the important role of the incorporation/presence of a heterocyclic ring (e.g., imidazole, piperazine, fluoro-isoquinoline, azole, and thiazole) on coumarin pharmacophore for antibacterial activity. The resulting coumarin derivatives had major activities—in particular, against the Gram-positive microorganisms. In addition, earlier researchers indicated the that free hydroxyl group at C-7 is crucial for antibacterial activity. The SAR study indicated that the choice of a suitable substitution containing electron-donating/withdrawing groups with certain heterocyclic derivatives in conjunction with a parent coumarin skeleton played a key role in the transformation of the antibacterial spectrum of synthesized derivatives. These compounds could be used as lead compounds for the development of future potent compounds. In addition, new coumarin conjugates were noted as dimers or in conjugation with other scaffolds with strong biological activity. Despite the new important findings, no new coumarins for such or other applications have been advanced to the clinical trial stage. The cellular mechanisms of coumarin derivatives are still unexplored, and there is a need for finding the molecular mechanism. It is also evident that their specificity is rather limited in the sense that some analogues possess wide activity profiles, often unpredictable and within similar concentration ranges. Further consideration towards the safety and toxicology studies of coumarin derivatives that are not yet completely available needs to be addressed. From this perspective, coumarins are not ready enough for the pharmaceutical industry, and further developments offer the chance to identify more novel therapeutics.

## Figures and Tables

**Table 1:** Patents on coumarin analogues as antimicrobial agents.

Patent Number	Patent Date	Description	References
CN103387582A	13 November 2013	Coumarin natural product, and preparation method and application thereof	[72]
CN102796085A	28 November 2013	Coumarin triazole, and preparation method and application thereof	[73]
WO2012137224A1	11 October 2012	Coumarin Compounds for The Treatment of Mycobacterial Infections	[74]
DE102010055566A1	21 June 2012	New compounds conjugating gyrase-inhibiting substances with catechol structural units, are gyrase inhibitors, useful as biologically active substances, and for treating bacterial infection	[75]
CN 105732601B	21 August 2018	Coumarin compound containing imidazolium-based functional groups and preparation method and application thereof	[76]
WO 2016096631A1	23 June 2016	Preparation of heterocyclic compounds as antibacterial agents	[77]
CN 105218501A	6 January 2016	Piperazine ring-containing coumarin derivatives and preparation method and application thereof in antibacterial drugs.	[78]
CN 106117232B	23 October 2018	Bacterial quorum-sensing inhibitor and antibacterial application thereof	[79]
CN 104829608B	30 June 2017	Preparation of coumarin thiazole-indolone type compound used for antibacterial drugs	[80]
CN 104910176A	10 May 2017	Coumarin azole compounds and preparation method and application thereof	[81]
CN 104817552A	27 June 2017	Preparation of (E)-N′- arylmethylene-4-(coumarin-3-yl) thiazole-2-hydrazide compounds as antimicrobial agents	[82]

## Data Availability

All data generated or analyzed during this study are included in this article.

## Abbreviations

CDCs, Centers for Disease Control and Prevention; DMSO, Dimethyl sulfoxide; DMF, Dimethylformamide; DCM, Dichloromethane; EtOH, Ethyl Alcohol; GAA, Glacial Acetic Acid; MDR, Multidrug Resistance; MRSA, Methicillin-resistant *Staphylococcus aureus*; MIC, Minimum Inhibitory Concentration; POCl_3_, Phosphoryl Chloride; SAR, Structural Activity Relationship; THF, Tetrahydrofuran; WHO, World Health Organization.
